# Increasing access to cognitive–behavioural therapy for patients with psychosis by evaluating the feasibility of a randomised controlled trial of brief, targeted cognitive–behavioural therapy for distressing voices delivered by assistant psychologists: the GiVE2 trial

**DOI:** 10.1192/bjo.2021.983

**Published:** 2021-08-19

**Authors:** Mark Hayward, Katherine Berry, Stephen Bremner, Anna-Marie Jones, Sam Robertson, Kate Cavanagh, Heather Gage, Clio Berry, Suzanne Neumann, Cassie M Hazell, David Fowler, Kathryn Greenwood, Clara Strauss

**Affiliations:** School of Psychology, University of Sussex, UK; and Research & Development Department, Sussex Partnership NHS Foundation Trust, UK; Faculty of Biology, Medicine & Health, Manchester Academic Health Sciences Centre, University of Manchester, UK; Brighton & Sussex Medical School, University of Sussex, UK; Research & Development Department, Sussex Partnership NHS Foundation Trust, UK; Research & Development Department, Sussex Partnership NHS Foundation Trust, UK; School of Psychology, University of Sussex, UK; School of Biosciences & Medicine, University of Surrey, UK; Brighton & Sussex Medical School, University of Sussex, UK; Research & Development Department, Sussex Partnership NHS Foundation Trust, UK; Social Sciences Department, University of Westminster, UK; School of Psychology, University of Sussex, UK; and Research & Development Department, Sussex Partnership NHS Foundation Trust, UK; School of Psychology, University of Sussex, UK; and Research & Development Department, Sussex Partnership NHS Foundation Trust, UK; School of Psychology, University of Sussex, UK; and Research & Development Department, Sussex Partnership NHS Foundation Trust, UK

**Keywords:** Voice hearing, psychosis, cognitive–behavioural therapy, feasibility, randomised controlled trial

## Abstract

**Background:**

Cognitive–behavioural therapy (CBT) is recommended for all patients with psychosis, but is offered to only a minority. This is attributable, in part, to the resource-intensive nature of CBT for psychosis. Responses have included the development of CBT for psychosis in brief and targeted formats, and its delivery by briefly trained therapists. This study explored a combination of these responses by investigating a brief, CBT-informed intervention targeted at distressing voices (the GiVE intervention) administered by a briefly trained workforce of assistant psychologists.

**Aims:**

To explore the feasibility of conducting a randomised controlled trial to evaluate the clinical and cost-effectiveness of the GiVE intervention when delivered by assistant psychologists to patients with psychosis.

**Method:**

This was a three-arm, feasibility, randomised controlled trial comparing the GiVE intervention, a supportive counselling intervention and treatment as usual, recruiting across two sites, with 1:1:1 allocation and blind post-treatment and follow-up assessments.

**Results:**

Feasibility outcomes were favourable with regard to the recruitment and retention of participants and the adherence of assistant psychologists to therapy and supervision protocols. For the candidate primary outcomes, estimated effects were in favour of GiVE compared with supportive counselling and treatment as usual at post-treatment. At follow-up, estimated effects were in favour of supportive counselling compared with GiVE and treatment as usual, and GiVE compared with treatment as usual.

**Conclusions:**

A definitive trial of the GiVE intervention, delivered by assistant psychologists, is feasible. Adaptations to the GiVE intervention and the design of any future trials may be necessary.

Approximately 300 000 people in England and Wales have a diagnosis of psychosis.^1^ The overall annual cost of psychosis in England has been estimated at £11.8 billion.^2^ Cognitive–behavioural therapy (CBT) for psychosis is recommended by the National Institute for Health and Care Excellence (NICE) for the treatment of psychosis,^3^ but is offered to only 26% of patients with psychosis.^4^ One reason for this lack of access is limited resources,^5^ as CBT for psychosis is resource-intensive in its duration (minimum of 16 sessions recommended by NICE) and delivery (by highly trained therapists, typically CBT therapists or clinical psychologists), and NICE has recommended research to explore how resources could be reduced.^3^ Our response has been twofold: first, we conducted a meta-analysis suggesting that CBT for psychosis could effectively treat the symptoms of psychosis when offered in fewer than 16 sessions;^6^ and second, consistent with recent developments favouring the targeting of single psychosis symptoms,^7^ we developed a brief, CBT-informed intervention for distressing voice hearing experiences (‘Guided self-help CBT intervention for voices’, referred to as GiVE). GiVE is a structured and workbook-based intervention^8^ derived from our self-help book,^9^ and has the potential to be delivered by a cost-effective and widely available workforce of psychology graduates (assistant psychologists). We initially evaluated GiVE when delivered by clinical psychologists, with promising results.^10^ Our next step was to explore the efficacy of GiVE when delivered by assistant psychologists. Before seeking funding for a definitive randomised controlled trial (RCT), we needed to demonstrate the feasibility of engaging patients, clinicians, assistant psychologists and service managers in the delivery of such a trial. The aims of this feasibility study were to assess the acceptability of GiVE to clinicians and patients, and the ability of assistant psychologists to adhere to therapy and clinical supervision protocols. An estimate of the standard deviation of outcomes was also sought to facilitate a sample size calculation for use within a definitive trial.

## Method

### Study design

The published study protocol^11^ is briefly described here. This was a feasibility RCT with a three-arm, parallel-group design and 1:1:1 allocation, comparing GiVE and treatment as usual (the study intervention) to supportive counselling and treatment as usual (the control intervention) to treatment as usual alone, recruiting across two sites, with blinded post-treatment and follow-up assessments. Clinical outcomes were assessed at baseline (pre-randomisation, time point 0), 16 weeks (post-intervention, time point 1) and 28 weeks (follow-up, time point 2; this assessment was only offered to participants who reached the 28-week post-randomisation milestone before the end of the data collection period). A mixed-methods process evaluation captured participants’, clinicians’ and assistant psychologists’ experiences of the study and the interventions, and their views on the facilitators and barriers to the implementation of GiVE within routine psychosis care pathways. Findings from the process evaluation will be reported in a separate paper. There were minimal changes to the design after trial commencement, and details are reported in the study protocol.^11^ The trial was registered with the ISRCTN registry (number 16166070) on 5 February 2019 (http://www.isrctn.com/ISRCTN16166070).

### Participants

Participants were recruited over 12 months from community mental health teams and early intervention in psychosis services at two sites within the UK National Health Service (NHS): Sussex Partnership NHS Foundation Trust and Pennine Care NHS Foundation Trust. Inclusion criteria were: aged 16 years or older in contact with NHS mental health services and under the care of a consultant psychiatrist; experiencing current voice hearing (a score of 1 or more [‘At least once a day’] on item 1 [‘How frequently did you hear a voice or voices?’] of the Hamilton Program for Schizophrenia Voices Questionnaire; HPSVQ);^11^ distressed by hearing voices (operationalised by participants scoring at least 8 out of 16 on the negative voice impact scale of the HPSVQ);^12^ meeting DSM-5 research criteria for schizophrenia spectrum or other psychotic disorders (assessed by the Structured Clinical Interview for DSM-5 disorders^13^); and willing and able to provide written informed consent. Exclusion criteria were: established organic cause for distressing voices (e.g. brain disease or injury); primary diagnosis of substance misuse; currently detained in hospital under a section of the Mental Health Act; have completed a full course (minimum of 16 h) of CBT for psychosis during the past year; currently participating, or confirmed to participate, in another interventional study in which the participant is receiving an intervention that utilises psychological therapy; English speaking to the degree that the participant is unable to fully understand and answer assessment questions and give informed consent; severe intellectual disability (assessed with the Test of Premorbid Functioning – UK)^14^; and deemed to be at immediate and serious risk to self or others.

### Procedure

Following a referral to the research team by a patient's clinician, a study research assistant contacted the potential participant to discuss the study and arrange a consent and eligibility meeting. A patient information sheet was given to the potential participant at least 24 h before the meeting. If participants consented to take part in the study, they completed a baseline (time point 0) assessment at a separate meeting.

The authors assert that all procedures contributing to this work comply with the ethical standards of the relevant national and institutional committees on human experimentation and with the Helsinki Declaration of 1975, as revised in 2008. All procedures involving patients were approved by an NHS Research Ethics Committee (London – Surrey, reference number 18/LO/ 2091). Written informed consent was obtained from all participants.

### Intervention description

#### GiVE

The GiVE intervention was delivered by an assistant psychologist and followed a workbook^8^ that was based upon the *Overcoming Distressing Voices* self-help book.^9^ Participants were given a copy of both the workbook and the self-help book at the commencement of therapy, and asked to read both and complete the workbook with the support of the assistant psychologist in sessions. They also had the opportunity to access the ‘Choices’ mobile phone application, a publicly available app with interactive elements linked to the content of the self-help book. GiVE consists of three core modules: beliefs about the self, beliefs about voices and relationships with voices and others. There is also an introductory session on coping with voices and a final session to consolidate learning and identify next steps. Assistant psychologists delivered GiVE within eight 1-h sessions, over a maximum of 16 weeks.

#### Supportive counselling

The supportive counselling intervention followed the therapy protocol that was used by assistant psychologists in the full RCT of AVATAR therapy.^15^ It was delivered over the same number and duration of sessions as GiVE, with the aim of equalising the duration of total therapist contact time across both arms. Supportive counselling offered a supportive and non-judgemental space for the discussion of topics and issues determined by the participant. The supportive counselling therapy protocol contained specific guidance for the assistant psychologist on how to respond to participant disclosure of distressing voices in a manner that did not provide specific intervention strategies. The intervention was delivered within eight 1-h sessions, over a maximum of 16 weeks. The same assistant psychologists delivered GiVE and supportive counselling, to minimise therapist effects.

#### Treatment as usual

Treatment as usual was provided by the usual care team and typically included medication management and support and monitoring, with psychological therapies offered occasionally.

### Training and supervision

Assistant psychologists received a 5-day training in GiVE and supportive counselling (1 introductory day, 2 days on GiVE and 2 days on supportive counselling), delivered by clinical psychologists with experience of CBT for psychosis and an experienced counselling psychologist, respectively. Weekly clinical supervision was provided by these clinical psychologists for the GiVE intervention, with a mixture of 1:1 and group supervision offered face to face or remotely. The counselling psychologist offered remote group supervision for the supportive counselling intervention on a weekly basis.

### Treatment adherence

Therapeutic drift and contamination were minimised by the use of highly detailed therapy protocols and close supervision of the assistant psychologists by experienced psychologists. Adherence to therapy protocols was assessed through assistant psychologists completing a checklist at the conclusion of each session. The competence of assistant psychologist delivery of the interventions was assessed by the rating of a random selection of session recordings by independent experts. GiVE sessions were rated by a CBT expert, using the Cognitive Therapy Rating Scale for Psychosis.^16^ Supportive counselling sessions were rated by a supportive counselling expert, using the Counselling Adherence Scale.^17^

### Outcomes

Feasibility outcomes prespecified in the published study protocol were as follows: number of care coordinators willing to refer their patients, number (percentage) of referred patients found to be eligible, number (percentage) of consenting participants retained within the study who offer full data-sets, proportion of non-missing items for each variable, number (percentage) of consenting participants within the GiVE and supportive counselling arms who reach the point of therapy ‘exposure’ (attend at least six out of eight therapy sessions), and assistant psychologist adherence to therapy protocols and clinical supervision protocols.

For primary outcomes for a definitive trial, two candidate measures were used to assess voice-related distress at time points 0, 1 and 2: the five-item distress scale of the Psychotic Symptom Rating Scales (PSYRATS auditory hallucinations subscale)^18^ (‘amount of negative voice content’, ‘degree of negative voice content’, ‘amount of distress’, ‘intensity of distress’ and ‘controllability of voices’) and the four-item negative voice impact scale of the HPSVQ^11^ (‘How bad are the things voices say to you?’, ‘How much do the voices interfere with your daily activities?’, ‘How distressing are the voices that you hear?’ and ‘How bad do the voices make you feel about yourself?’).

A range of secondary outcomes were assessed at time points 0, 1 and 2, and evaluated mental health problems commonly experienced by people with psychosis, including anxiety and depression (Hospital Anxiety and Depression Scale^19^) and paranoia (Paranoid Thoughts Scale^20^); variables that have been associated with the impact of voice hearing, such as negative beliefs about the self (Self Scale of the Brief Core Schema Scale)^21^, negative beliefs about voices (Beliefs About Voices Questionnaire – Revised^22^) and negative relating to voices (Voice and You^23^) and other people (Persons Relating to Others Questionnaire^24^); and a range of personal and social recovery-oriented outcomes (psychological recovery [CHOICE-SF],^25^ the positive impact of voices [Voice Impact Scale] (Strauss C, personal communication, 2018), engagement in meaningful activity [Work and Social Adjustment Scale]^26^ and social functioning [Social and Occupational Functioning Scale]^27^).

We assessed participants’ expectations for therapy following the commencement of the intervention between time point 0 and time point 1 (Therapy Credibility and Expectancy Questionnaire)^28^ and the quality of the therapeutic relationships at time point 1 (Scale to Assess Therapeutic Relationships).^29^

Health economic outcomes were measured by the EQ-5D-5L,^30^ the Client Service Receipt Inventory^31^ and the Short Form 12 Version 2.^32^

### Sample size

Following recommendations for designing feasibility trials that aim to detect a small-to-medium (Cohen's *d* = 0.2–0.5) standardised effect size (where the definitive trial will be designed with 90% power and two-sided 5% significance), this study aimed to recruit 90 participants (30 per treatment arm).^33–36^

### Randomisation and blinding

Following the completion of the baseline assessment, participants were randomly allocated by the trial manager using the Sealed Envelope online service (https://www.sealedenvelope.com/), incorporating stratification by site (Sussex or Pennine) and type of service (community mental health team or early intervention in psychosis) by using random block lengths and 1:1:1 allocation. The online randomisation procedure was set up and tested by the trial statistician.

The research assistants were blind to the allocation sequence and remained so for all future assessments. Measures were put in place to maintain blinding, and ‘blind’ awareness and education was promoted throughout the study. The trial statistician was also blind to participant allocation throughout the analysis. Reported breaks in blinding were recorded. Outcome assessments were re-blinded by re-allocating blind research assistants to collect and score participant responses.

### Statistical analysis

Participant characteristics, feasibility outcomes and baseline clinical outcomes were summarised with descriptive statistics such as count, mean, s.d., median, 25% and 75% quartiles, interquartile range, minimum and maximum. Analyses were of available cases, following the intention-to-treat principle. All clinical outcomes were summarised at time points 0, 1 and 2 for each treatment arm. Primary and secondary outcomes were modelled with linear mixed models (to allow for covariate adjustment), with random effects for individuals and fixed effects for treatment group (treatment as usual, GiVE and supportive counselling), time (time points 1 and 2) and the group×time interaction with the baseline score, site and service type entered as covariates. Contrasts were used for the following comparisons: GiVE versus treatment as usual, GiVE versus supportive counselling, and supportive counselling versus treatment as usual at time points 1 and 2. 95% and 75% confidence intervals for all estimates of unstandardised between-group effect sizes were calculated. For each contrast we were interested in whether the minimum clinically important difference (MCID) was contained in the confidence interval. The MCIDs are two points for the HPSVQ negative voice impact scale and three points for the PSYRATS distress scale. Standardised effect sizes (Cohen's *d*) were also calculated and interpreted as follows: 0.2 = small, 0.5 = medium and 0.8 = large effect.^33^ As this was a feasibility study, *P*-values from the models are not reported. The level of missing data was summarised at each time point, but not treated. All analyses were conducted in Stata (version 16 for Mac).^37^

### Adverse event reporting and harms

Any unfavourable and unintended sign, symptom or illness that developed or worsened during the period of the study was classified as an adverse event, whether or not it was considered to be related to the study treatment and was either expected or unexpected. Serious adverse events (SAEs) were those considered to be life-threatening. The number (events and individuals) and nature of all events (adverse events and SAEs) reported to blind and unblind members of the research team were recorded. All SAEs were reviewed for causality and expectedness by an independent rater and the sponsor's representative.

### Impact of the COVID-19 pandemic

The onset of the pandemic in March 2020 required all assessments and therapy sessions to be delivered remotely by phone or video call. At time point 1, 21 out of 68 (31%) assessments were completed remotely. For time point 2, this figure was 15 out of 46 (33%) assessments. Regarding intervention delivery, 11 participants who were receiving sessions face to face at the onset of the pandemic were offered remote sessions (seven for GiVE and four for supportive counselling), and a further two participants (one for GiVE and one for supportive counselling) received all sessions remotely.

## Results

### Participant flow

**Fig. 1:**
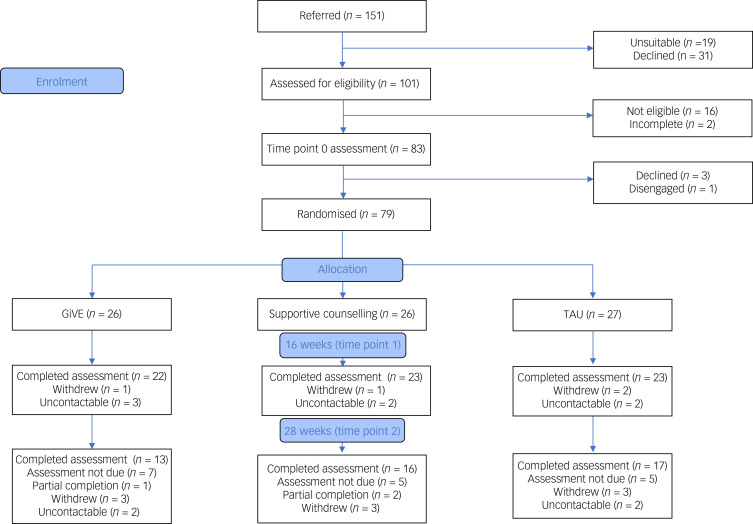
CONSORT flow diagram.

A total of 151 patients were referred to the study over a 12-month period between 13 February 2019 and 24 February 2020. A total of 101 (66.9%) patients were screened, of whom 79 (78.2%) consented, completed a time point 0 assessment and were randomised as follows: 27 to treatment as usual, 26 to supportive counselling and 26 to GiVE. Time point 1 and time point 2 assessments were completed between July 2019 and July 2020. See Fig. 1 for CONSORT diagram illustrating the flow of participants through the study. The study was concluded as planned, on 30 August 2020. The final 6 months of the study were conducted during the COVID-19 pandemic.

### Participant characteristics

There were 45 (57%) male and 34 (43%) female participants, with a mean age of 40.1 years (s.d. 13.1, range 17–66) and, on average, had been hearing voices since the age of 22.0 years (s.d. 12.1, 0.1–61). Mean summary scores for the baseline candidate primary outcomes were 11.6 (s.d. 2.5) for the HPSVQ negative voice impact scale and 14.5 (s.d. 3.3) for the PSYRATS distress scale. All baseline participant characteristics and clinical outcomes are shown in Table 1.

**Table 1:** Baseline characteristics by treatment group

	Treatment as usual (*n* = 27)	Guided self-help intervention for voices (*n* = 26)	Supportive counselling (*n* = 26)
Age, years	27; 42.3 (13)	26; 39.6 (11)	26; 38.3 (15)
Gender			
Male	19 (70%)	15 (58%)	11 (42%)
Female	8 (30%)	11 (42%)	15 (58%)
Ethnicity			
White British/White other	23 (85%)	23 (88.5%)	20 (77%)
Black, Asian and minority ethnic	3 (11%)	3 (11.5%)	4 (15%)
Other	1 (4%)	0 (0%)	2 (8%)
Marital status			
Single/separated/divorced	24 (89%)	18 (69%)	20 (81%)
Married/civil partnership/ cohabiting	3 (11%)	8 (31%)	5 (19%)
Employed			
Yes	2 (7%)	2 (8%)	1 (4%)
No	25 (93%)	24 (92%)	25 (96%)
Highest education attainment*			
None	4 (15%)	8 (31%)	5 (19%)
Secondary school/high school (GCSE or equivalent	7 (26%)	7 (27%)	11 (42%)
College/sixth form	7 (26%)	5 (19%)	5 (19%)
Undergraduate/postgraduate	5 (19%)	5 (19%)	2 (8%)
Other	4 (15%)	1 (4%)	3 (12%)

Age at onset of voice hearing, years	27; 22 (11)	26; 22 (13)	26; 23 (13)
Outcome measures			
HPSVQ: negative voice impact (0–16)	27; 11.6 (2.8)	26; 11.6 (2.2)	26; 11.7 (2.4)
PSYRATS: distress (0–20)	27; 14.8 (3.0)	26; 14.8 (2.8)	26; 13.8 (4.1)
HADS: depression (0–21)	27; 11.7 (3.5)	26; 11.7 (3.6)	26; 8.4 (3.4)
HADS: anxiety (0–21)	27; 13.9 (3.8)	26; 13.8 (3.6)	26; 11.6 (4.4)
CHOICE-SF: mean (0–10)	27; 4.0 (1.3)	26; 3.9 (1.6)	26; 4.5 (1.7)

Data are count *n* (%), mean (s.d.) or *n*; mean (s.d.). Numbers in brackets after name of the measure is the range of possible values; clinical outcomes are normally distributed. HPSVQ, Hamilton Program for Schizophrenia Voices Questionnaire; PSYRATS, Psychotic Symptoms Rating Scale; HADS, Hospital Anxiety and Depression Scale; CHOICE-SF, Choice in Outcome in CBT for Psychoses, Short Form; * some percentage totals may add up to more than 100% due to rounding error.

### Feasibility outcomes

#### Aim 1: to assess the acceptability to clinicians of the GiVE intervention delivered by assistant psychologists (will they refer patients to the study?)

Patients were referred to the study by 102 different clinicians (50 from the Pennine site and 52 from the Sussex site). Of the 101 patients who consented and were assessed for eligibility, 83 (82%, 95% CI 73.3–89.1%) were found to be eligible. The views of some of the referring clinicians were explored within the process evaluation and will be reported in a separate paper.

#### Aim 2: to assess the acceptability to patients of the GiVE intervention delivered by assistant psychologists (can patients be recruited and retained, and what are their experiences of the intervention?)

We randomised 79 (88%) of our target of 90 participants. Of those randomised, 68 (86%) participants completed time point 1 assessments and 46 (74%) participants who reached the 28-week post-randomisation milestone completed time point 2 assessments.

In terms of therapy exposure, 23 (88%) participants attended at least six sessions in the GiVE arm (the predetermined level of exposure), and this was the case for 19 (73%) participants in the supportive counselling arm. The experiences of some of the participants who received the GiVE and supportive counselling interventions were explored within the process evaluation and will be reported in a separate paper.

#### Aim 3: to assess the ability of assistant psychologists to adhere to the therapy and clinical supervision protocols

All attended sessions of GiVE were self-rated for adherence by assistant psychologists, using therapy checklists, and adherence was indicated for 83% of items. There was some variability by therapist (range 72–97%) and by session (range 70–88%). For supportive counselling, all but one attended session was rated by assistant psychologists and adherence was indicated for 95% of items. There was some variability in adherence by therapist (range 89–100%) and by session (range 92–97%).

With regard to assistant psychologist competence, the independent raters were external to the NHS organisations that were hosting the trial. Changes to the policies of the NHS organisations relating to the transfer of patient identifying information created complexities for the secure transfer of the audio recordings outside of these organisations. We were not able to overcome these complexities before the end of the trial. Additionally, during the COVID-19 pandemic it was not possible to record sessions delivered remotely because of home working. At the Sussex site, 58 GiVE sessions were recorded (out of a total of 185 sessions delivered; 31%) and 50 supportive counselling sessions were recorded (out of a total of 162 sessions delivered; 31%). Six audio recordings of randomly selected GiVE sessions (3%) from the Sussex site were independently rated with the Cognitive Therapy Rating Scale – Psychosis. The mean was 31.7 (s.d. 3.8) out of a maximum of 60. The mean score for general skills was 17.5 (s.d. 3.1) out of a maximum of 30. The mean score for technical skills was 14.2 (s.d. 2.1) out of a maximum of 30. The ratings revealed that although assistant psychologists demonstrated skills in general understanding and interpersonal skills, they were less able to link sessions though homework and feedback. Ratings were completed for five supportive counselling sessions (3%) using the Counselling Adherence Scale, and the mean was 17⋅8 (s.d. 2.4) out of a maximum of 28, which equates to moderate-to-good evidence of competence. To assess for drift between the interventions, the supportive counselling session recordings were assessed by the independent CBT rater against the session checklists for the GiVE intervention. For three of the recordings, the rater found ‘no evidence’ of items from the GiVE checklists. On the remaining two recordings, there was ‘negligible evidence’ of items from the GiVE checklists. These assessments suggest that any drift from the GiVE intervention to the supportive counselling intervention was minimal.

Adherence to clinical supervision protocols was assessed through the recording of attendance at weekly supervision sessions for each of the four assistant psychologists. Attendance rates were as follows: 89.0%, 91.6%, 96.4% and 98.0%.

### Completeness of data

Items were mostly at least 90% complete, except for the Scale to Assess Therapeutic Relationships - Patient version (missing data was 79% owing to measures not being posted back by participants), the Therapy Credibility and Expectancy Questionnaire (missing data was 25%), the Short Form 12 Version 2 (missing data was 53% at time point 0, 92% at time point 1 and 100% at time point 2 [owing to administration errors]), and some of the sociodemographic characteristics.

### Candidate primary outcomes

The descriptive summary for the candidate primary and selected secondary outcomes at time points 1 and 2 are shown in Table 2.

**Table 2:** Summary of candidate primary (HPSVQ & PSYRATS) and secondary clinical outcomes by group at time points 1 and 2

	Clinical outcome (range)	Treatment as usual (*n* = 27 randomised), *n*; mean (s.d.)	Guided self-help intervention for voices (*n* = 26 randomised), *n*; mean (s.d.)	Supportive counselling (*n* = 26 randomised), *n*; mean (s.d.)
Time point 1	HPSVQ: negative voice impact (0–16)	23; 10.2 (4.6)	22; 8.1 (4.0)	23; 10.0 (4.1)
	PSYRATS: distress (0–20)	23; 13.6 (4.7)	22; 11.8 (5.4)	22; 14.0 (4.2)
	HADS: depression (0–21)	23; 11.1 (4.9)	22; 8.9 (3.6)	23; 8.6 (4.6)
	HADS: anxiety (0–21)	23; 13.1 (4.1)	22; 11.9 (4.4)	23; 11.6 (4.0)
	CHOICE-SF: mean (0–10)	23; 3.9 (1.8)	22; 5.6 (2.1)	23; 5.2 (2.2)
Time point 2	HPSVQ: negative voice impact (0–16)	14; 9.5 (2.3)	12; 9.2 (5.0)	17; 8.4 (5.2)
	PSYRATS: distress (0–20)	15; 14.3 (2.4)	12; 13.0 (4.8)	16; 12.4 (4.3)
	HADS: depression (0–21)	15; 10.5 (3.8)	12; 10.0 (5.0)	16; 7.4 (4.4)
	HADS: anxiety (0–21)	15; 11.9 (4.4)	12; 11.3 (4.2)	16; 10.0 (5.9)
	CHOICE-SF: mean (0–10)	15; 4.2 (1.7)	12; 4.8 (2.5)	16; 5.6 (2.7)

HPSVQ, Hamilton Program for Schizophrenia Voices Questionnaire; PSYRATS, Psychotic Symptoms Rating Scale; HADS, Hospital Anxiety and Depression Scale; CHOICE-SF, Choice in Outcome in CBT for Psychoses, Short Form.

Results from the intention-to-treat analyses of the candidate primary outcomes are shown in Table 3. Details regarding the secondary outcomes and the health economic outcomes are provided in the Supplementary material available at https://doi.org/10.1192/bjo.2021.983.

**Table 3:** Effect sizes for candidate primary outcomes by time point

Clinical outcome	Pairwise comparison	Time point 1					Time point 2				
		Effect estimate	s.e.	95% LCL	95% UCL	Cohen's *d*^a^	Effect estimate	s.e.	95% LCL	95% UCL	Cohen's *d*^a^
HPSVQ: negative voice impact	GiVE versus TAU	−1.75	1.13	−3.96	0.46	−0.71	−0.37	1.37	−3.06	2.32	−0.15
	Supportive counselling versus TAU	0.08	1.11	−2.09	2.25	0.03	−1.01	1.28	−3.51	1.50	−0.41
	GiVE versus supportive counselling	−1.83	1.12	−4.02	0.36	−0.75	0.64	1.33	−1.96	3.24	0.26
PSYRATS: distress	GiVE versus TAU	−1.63	1.19	−3.97	0.72	−0.49	−1.66	1.45	−4.50	1.18	−0.50
	Supportive counselling versus TAU	0.92	1.21	−1.45	3.29	0.28	−1.44	1.38	−4.14	1.27	−0.44
	GiVE versus supportive counselling	−2.54	1.20	−4.90	−0.19	−0.77	−0.22	1.43	−3.03	2.59	−0.07

Table provides results of pairwise comparisons of intervention *[A]* versus *[B]* at time point 1/time point 2. Effect estimates represent a difference in change for *[A]* compared with *[B]*: negative scores indicate that *[A]* has improved more and positive scores indicate that *[B]* has improved more. For Cohen's *d* calculations, HPSVQ s.d. = 2.46 and PSYRATS s.d. = 3.30. LCL, lower confidence limit; UCL, upper confidence limit; HPSVQ, Hamilton Program for Schizophrenia Voices Questionnaire; GiVE, guided self-help intervention for voices; TAU, treatment as usual; PSYRATS, Psychotic Symptoms Rating Scale.

a. Conventions for interpreting Cohen's *d* are as follows: 0.2 = small effect, 0.5 = medium effect and 0.8 = large effect.^33^

All 95% confidence intervals included their respective outcome's MCID. For the candidate primary outcomes at time point 1, estimated effects were in favour of GiVE compared with both supportive counselling and treatment as usual. For these comparisons, Cohen's *d* standardised effect sizes ranged from medium to large. At time point 2 for HPSVQ negative voice impact: estimated effects were in favour of supportive counselling compared with both GiVE (small effect) and treatment as usual (small to medium effect); effects for GiVE were favourable compared with treatment as usual (small effect). For PSYRATS distress at time point 2: estimated effects were in favour of GiVE compared with both supportive counselling (negligible effect) and treatment as usual (medium effect); supportive counselling was favourable to treatment as usual (small to medium effect).

### Breaks in blinding

One break in blinding was reported. In this instance, the assessment was completed by a blinded researcher who was not part of the research team.

### Assessment of safety

SAEs were reported by 11 participants on a total of 16 occasions. The most frequently recorded SAE code was ‘suicidal ideation and intent to act’ (3). The SAEs were distributed evenly across the three arms. Independent assessment suggested that no SAEs were related to the trial or its procedures. Adverse events were reported by 20 participants on a total of 30 occasions. The most frequently recorded adverse event codes were ‘deterioration in mental health’ (*n* = 10), ‘general medical assessment/procedure’ (*n* = 8), ‘suicidal ideation’ (*n* = 6) and ‘self-harm’ (*n* = 4). The adverse events were evenly distributed across the three arms.

## Discussion

This study has demonstrated that a definitive RCT of the GiVE intervention delivered by assistant psychologists to patients with psychosis is feasible. Clinicians were willing to refer their patients, the majority of participants completed interventions and were retained at follow-up, and assistant psychologists were adherent to therapy and supervision protocols. The interventions appeared to be safe and the MCIDs for the candidate primary outcomes were captured in the 95% confidence intervals for the between-group differences in the means at time point 1.

The favourable levels of referrals and recruitment suggest a willingness on behalf of clinicians and patients with psychosis to engage with a trial when the intervention is delivered by briefly trained assistant psychologists. Once randomised, participants demonstrated further willingness to engage in the study by attending therapy sessions and time point 1 assessments. The level of therapy exposure was highest for the participants receiving the GiVE intervention (88%), with an exposure rate higher than comparable trials where CBT for psychosis was delivered by highly trained therapists (e.g. 72%^38^). Attendance at time point 2 assessments was lower than at time point 1. Although noteworthy, attempts were made to engage only a subset of participants (as planned) in the time point 2 assessments, and the limited attendance may, in part, be attributable to the COVID-19 pandemic, which led to all assessments being conducted remotely during the final 6 months of data collection.

Willingness to engage with the trial was also required by the other important stakeholder group – the assistant psychologists. To our knowledge, no studies have previously explored the adherence of this workforce to therapy and supervision protocols when working with patients with psychosis. Consequently, rigorous assessment was required. Findings were encouraging, with each of the assistant psychologists attending most of the weekly clinical supervision sessions offered. Self-rated adherence to the therapy protocols was also high, but with variation across particular sessions indicating areas for additional training within a future trial. The rating of competence was restricted by the limited availability of session recordings as a result of information governance issues and the COVID-19 pandemic. The former issues will need to be addressed before a future trial. The available ratings suggested that assistant psychologists could achieve competence in the delivery of both GiVE and supportive counselling. However, the ratings for the GiVE intervention suggested some areas for improvement, including the linking of learning during and between sessions, which could be addressed in a future trial with specific training and supervision on this issue. The assessment of contamination between the interventions suggested that the assistant psychologists had been able to prevent the GiVE techniques from drifting into the supportive counselling sessions.

Findings were also encouraging with respect to the candidate primary outcomes. Medium-to-large estimated effects were found in favour of GiVE compared with both supportive counselling and treatment as usual at time point 1. The effect sizes were in line with prior expectations of detecting small-to-medium effects with some inflation owing to the small sample size. Although effects detected in small feasibility studies, such as this one, are likely to be imprecise and biased, the size and magnitude of our detected effects support further testing in a powered RCT. The findings at time point 2 were less clear as the estimated effects favoured supportive counselling compared with GiVE and treatment as usual with (negligible-small and medium effects, respectively). However, GiVE was still favourable compared with treatment as usual negligible-medium effects at time point 2. The findings at time point 2 were attributable, in part, to a reduction of benefits for GiVE during the follow-up period (from time point 1 to time point 2), and may indicate the need for the intervention to be extended and/or for the addition of booster sessions. This reduction in benefit for GiVE during the follow-up period also raises the question of the potential place of this intervention within a therapeutic pathway for patients with psychosis, e.g. as part of a modular pathway where other single-symptom interventions could build upon the learning generated by GiVE, or *vice versa*. A modular pathway for the psychological treatment of paranoia has recently been evaluated and generated encouraging findings, albeit with the interventions delivered by highly trained therapists.^39^ The findings of increased benefits for supportive counselling during the follow-up period are similar to those reported by the recently completed RCT of AVATAR therapy.^14^ Those authors suggested that supportive counselling is a control condition with non-specific factors that, compared with treatment as usual, can be effective in its own right. This is a possibility that is worthy of further investigation.

This study has limitations in a number of respects. First, the follow-up data were available for only a subsample of the participants (as planned), thereby limiting the learning about the durability of benefits from the interventions. Second, the self-reported adherence ratings of the assistant psychologists were not assessed and would benefit from independent assessment within a future trial. Furthermore, the competence ratings of the assistant psychologists were limited to a small selection of session recordings. Further learning about the competence of assistant psychologists within a future trial will be crucial, and any barriers to the rating of competence must be addressed *a priori*. Finally, the findings may have been influenced by the COVID-19 pandemic. Some of the therapy sessions and assessments were conducted remotely during the final 6 months of the study, but any impact of remote delivery and/or the more general impact of the pandemic upon participants could not be established because of the small sample size.

The main implication of this trial is that a definitive RCT is feasible and is now required to provide evidence regarding the efficacy of a brief, CBT-informed intervention for distressing voices when delivered to patients with psychosis by assistant psychologists. If found to be efficacious in future trials, CBT for psychosis offered in these less resource-intensive forms has the potential to generate benefits for individual patients (reduced distress and enhanced recovery), service-level patient benefits (increased access to evidence-based psychological therapies) and economic benefits to the NHS (in terms of the reduced use of high-impact mental health services).

## Data Availability

The data that support the findings of this study are available from the corresponding author, M.H., upon reasonable request.

## Supplementary material

For supplementary material accompanying this paper visit http://dx.doi.org/10.1192/bjo.2021.983. click here to view supplementary material
