# Supplementary material for: Increasing access to cognitive–behavioural therapy for patients with psychosis by evaluating the feasibility of a randomised controlled trial of brief, targeted cognitive–behavioural therapy for distressing voices delivered by assistant psychologists: the GiVE2 trial
Source: BJPsych Open. 2021 Aug 19;7(5):e152. doi: 10.1192/bjo.2021.983 (PMC8388007; doi:10.1192/bjo.2021.983)
Supplement: Supplementary file 1 [file bjosup.zip › S2056472421009832sup001.docx]

**Supplementary Information - Tables**

Contents

Supplementary Table 1: Descriptive summary of Secondary Outcomes by Group and Time point Page 2

Supplementary Table 2: Effect estimates for Primary & Secondary Clinical Outcomes by time point Page 19

**Supplementary Table 1: Descriptive summary of Secondary Outcomes by Group and Time point**

| **Time point & Group** | **Outcome** | **Score Possible Range** | **Normally Distributed** | **N** | **mean** | **sd** | **25^th^ centile** | **median** | **75^th^ centile** | **IQR** | **min** | **max** |
| --- | --- | --- | --- | --- | --- | --- | --- | --- | --- | --- | --- | --- |
| T0 | HPSVQ: Voice Characteristics | 0-20 | Yes | 27 | 13.5 | 2.9 | 12 | 14 | 16 | 4 | 5 | 18 |
| TAU | PSYRATS: Frequency | 0-12 | Yes | 27 | 7 | 1.86 | 6 | 7 | 8 | 2 | 3 | 10 |
|  | PSYRATS: Loudness | 0-4 | Yes | 27 | 2.52 | 1.05 | 2 | 2 | 4 | 2 | 1 | 4 |
|  | PSYRATS: Attribution | 0-8 | Yes | 26 | 4.73 | 1.61 | 4 | 4.5 | 6 | 2 | 2 | 8 |
|  | PSYRATS: Total | 0-44 | Yes | 26 | 29.2 | 5.32 | 25 | 28.5 | 33 | 8 | 17 | 37 |
|  | VIS: Negative Impact | 0-80 | No | 27 | 15.9 | 11 | 7 | 16 | 25 | 18 | 0 | 45 |
|  | VIS: Positive Impact | 0-80 | No | 27 | 11.6 | 15.2 | 0 | 4 | 27 | 27 | 0 | 48 |
|  | VIS: Living Well | 0-80 | Yes | 27 | 34.6 | 15.3 | 25 | 32 | 43 | 18 | 4 | 63 |
|  | VIS: Total | 0-240 | Yes | 27 | 62.1 | 30.1 | 45 | 64 | 77 | 32 | 4 | 120 |
|  | HADS: Depression | 0-21 | Yes | 27 | 11.7 | 3.54 | 8 | 12 | 14 | 6 | 6 | 17 |
|  | HADS: Anxiety | 0-21 | Yes | 27 | 13.9 | 3.78 | 10 | 13 | 17 | 7 | 9 | 21 |
|  | CHOICE-SF: Mean | 0-10 | Yes | 27 | 4.02 | 1.3 | 2.91 | 4.09 | 4.82 | 1.91 | 2.09 | 6.55 |
|  | CHOICE-SF: Goal Rating | 0-10 | Yes | 27 | 2.59 | 2.32 | 1 | 2 | 4 | 3 | 0 | 9 |
|  | BCSS: Negative Self | 0-24 | No | 26 | 10.5 | 5.61 | 7 | 10 | 14 | 7 | 1 | 21 |
|  | BCSS: Positive Self | 0-24 | No | 27 | 5.93 | 4.97 | 2 | 4 | 12 | 10 | 0 | 16 |
|  | VAY: Voice Dominance | 0-21 | No | 27 | 16.3 | 5.33 | 12 | 19 | 21 | 9 | 3 | 21 |
|  | VAY: Voice Intrusiveness | 0-15 | No | 27 | 9.26 | 4 | 6 | 9 | 13 | 7 | 0 | 15 |
|  | VAY: Hearer Dependence | 0-27 | No | 27 | 7.07 | 4.16 | 4 | 7 | 8 | 4 | 0 | 19 |
|  | VAY: Hearer Distance | 0-21 | No | 27 | 16.0 | 4.59 | 12 | 18 | 20 | 8 | 6 | 21 |
|  | BAVQ: Persecutory | 0-27 | Yes | 27 | 14.4 | 6.2 | 9 | 15 | 20 | 11 | 3 | 25 |
|  | BAVQ: Benevolence | 0-15 | No | 27 | 2.26 | 3.32 | 0 | 0 | 4 | 4 | 0 | 11 |
|  | PROQ3: UN Negative | 0-15 | No | 27 | 5.96 | 4.05 | 3 | 5 | 9 | 6 | 0 | 15 |
|  | PROQ3: UN Positive | 0-3 | No | 27 | 1.22 | 1.34 | 0 | 1 | 3 | 3 | 0 | 3 |
|  | PROQ3: UC Negative | 0-15 | No | 27 | 3.74 | 3.81 | 1 | 3 | 4 | 3 | 0 | 15 |
|  | PROQ3: UC Positive | 0-3 | No | 27 | 2.33 | 1.04 | 2 | 3 | 3 | 1 | 0 | 3 |
|  | PROQ3: NC Negative | 0-15 | No | 27 | 5.67 | 4.88 | 1 | 5 | 9 | 8 | 0 | 15 |
|  | PROQ3: NC Positive | 0-3 | No | 27 | 1.26 | 1.29 | 0 | 1 | 3 | 3 | 0 | 3 |
|  | PROQ3: LC Negative | 0-15 | No | 27 | 10.1 | 3.47 | 8 | 10 | 13 | 5 | 3 | 15 |
|  | PROQ3: LC Positive | 0-3 | No | 27 | 2.19 | 0.96 | 1 | 3 | 3 | 2 | 0 | 3 |
|  | PROQ3: LN Negative | 0-15 | No | 27 | 8 | 3.78 | 6 | 9 | 10 | 4 | 0 | 15 |
|  | PROQ3: LN Positive | 0-3 | No | 27 | 1.93 | 1.17 | 1 | 2 | 3 | 2 | 0 | 3 |
|  | PROQ3: LD Negative | 0-15 | No | 27 | 8.67 | 3.84 | 6 | 8 | 12 | 6 | 3 | 15 |
|  | PROQ3: LD Positive | 0-3 | No | 27 | 1.74 | 1.16 | 1 | 2 | 3 | 2 | 0 | 3 |
|  | PROQ3: ND Negative | 0-15 | No | 27 | 11.2 | 3.84 | 8 | 13 | 15 | 7 | 3 | 15 |
|  | PROQ3: ND Positive | 0-3 | No | 27 | 2.3 | 0.95 | 2 | 3 | 3 | 1 | 0 | 3 |
|  | PROQ3: UD Negative | 0-15 | No | 27 | 5.22 | 4.21 | 2 | 3 | 9 | 7 | 0 | 13 |
|  | PROQ3: UD Positive | 0-3 | No | 27 | 1.26 | 1.32 | 0 | 1 | 3 | 3 | 0 | 3 |
|  | PROQ3: Total | 0-144 | Yes | 27 | 72.8 | 19.8 | 61 | 69 | 86 | 25 | 36 | 117 |
|  | PTS: Ideas of Reference | 16-80 | No | 27 | 52.3 | 13.9 | 43 | 56 | 61 | 18 | 20 | 76 |
|  | PTS: Ideas of Persecution | 16-80 | No | 27 | 52.8 | 17.9 | 38 | 55 | 67 | 29 | 16 | 79 |
|  | WSNS: Total | 0-40 | Yes | 27 | 28.7 | 5.77 | 23 | 29 | 33 | 10 | 20 | 40 |
|  | SOFAS: Total | 0-100 | Yes | 26 | 41 | 9.83 | 33 | 40 | 50 | 17 | 20 | 59 |
|  |  |  |  |  |  |  |  |  |  |  |  |  |
| **Time point & Group** | **Outcome** | **Score Possible Range** | **Normally Distributed** | **N** | **mean** | **sd** | **25^th^ centile** | **median** | **75^th^ centile** | **IQR** | **min** | **max** |
| T0 | HPSVQ: Voice Characteristics | 0-20 | Yes | 26 | 13.2 | 2.37 | 12 | 13 | 14 | 2 | 9 | 19 |
| GiVE | PSYRATS: Frequency | 0-12 | Yes | 26 | 7.23 | 1.84 | 6 | 7 | 9 | 3 | 4 | 10 |
|  | PSYRATS: Loudness | 0-4 | Yes | 26 | 2.08 | 0.85 | 2 | 2 | 2 | 0 | 1 | 4 |
|  | PSYRATS: Attribution | 0-8 | Yes | 26 | 4.23 | 1.66 | 3 | 4 | 5 | 2 | 2 | 8 |
|  | PSYRATS: Total | 0-44 | Yes | 26 | 28.4 | 4.52 | 26 | 28.5 | 31 | 5 | 19 | 36 |
|  | VIS: Negative Impact | 0-80 | No | 26 | 17.7 | 12.3 | 8 | 16.5 | 26 | 18 | 0 | 43 |
|  | VIS: Positive Impact | 0-80 | No | 25 | 10.8 | 11.9 | 0 | 5 | 24 | 24 | 0 | 35 |
|  | VIS: Living Well | 0-80 | Yes | 26 | 36.7 | 13.6 | 26 | 36.5 | 48 | 22 | 4 | 62 |
|  | VIS: Total | 0-240 | Yes | 25 | 64.6 | 27.5 | 43 | 64 | 87 | 44 | 21 | 112 |
|  | HADS: Depression | 0-21 | Yes | 26 | 11.7 | 3.61 | 10 | 12 | 14 | 4 | 4 | 21 |
|  | HADS: Anxiety | 0-21 | Yes | 26 | 13.8 | 3.63 | 11 | 14 | 16 | 5 | 7 | 21 |
|  | CHOICE-SF: Mean | 0-10 | Yes | 26 | 3.87 | 1.62 | 2.55 | 3.77 | 5 | 2.45 | 1 | 6.73 |
|  | CHOICE-SF: Goal Rating | 0-10 | Yes | 26 | 3.54 | 2.83 | 1 | 3 | 5 | 4 | 0 | 10 |
|  | BCSS: Negative Self | 0-24 | No | 26 | 7.54 | 5.85 | 3 | 6 | 12 | 9 | 0 | 20 |
|  | BCSS: Positive Self | 0-24 | No | 26 | 7.58 | 4.6 | 4 | 8 | 10 | 6 | 0 | 18 |
|  | VAY: Voice Dominance | 0-21 | No | 26 | 15.3 | 4.36 | 12 | 16 | 19 | 7 | 7 | 21 |
|  | VAY: Voice Intrusiveness | 0-15 | No | 26 | 9.69 | 3.32 | 6 | 11 | 12 | 6 | 4 | 15 |
|  | VAY: Hearer Dependence | 0-27 | No | 26 | 7.04 | 5.52 | 3 | 6 | 8 | 5 | 0 | 22 |
|  | VAY: Hearer Distance | 0-21 | No | 26 | 16.1 | 3.95 | 13 | 17 | 19 | 6 | 9 | 21 |
|  | BAVQ: Persecutory | 0-27 | Yes | 26 | 14.5 | 5.72 | 10 | 13 | 20 | 10 | 5 | 25 |
|  | BAVQ: Benevolence | 0-15 | No | 26 | 1.38 | 2.32 | 0 | 0 | 2 | 2 | 0 | 7 |
|  | PROQ3: UN Negative | 0-15 | No | 26 | 6.04 | 4.02 | 3 | 5.5 | 9 | 6 | 0 | 13 |
|  | PROQ3: UN Positive | 0-3 | No | 26 | 1.19 | 1.2 | 0 | 1 | 2 | 2 | 0 | 3 |
|  | PROQ3: UC Negative | 0-15 | No | 26 | 3.58 | 2.7 | 2 | 3 | 6 | 4 | 0 | 10 |
|  | PROQ3: UC Positive | 0-3 | No | 26 | 2.54 | 0.99 | 3 | 3 | 3 | 0 | 0 | 3 |
|  | PROQ3: NC Negative | 0-15 | No | 26 | 6 | 3.98 | 3 | 6 | 9 | 6 | 0 | 15 |
|  | PROQ3: NC Positive | 0-3 | No | 26 | 1.85 | 1.35 | 0 | 3 | 3 | 3 | 0 | 3 |
|  | PROQ3: LC Negative | 0-15 | No | 26 | 8.77 | 4.75 | 4 | 9 | 13 | 9 | 0 | 15 |
|  | PROQ3: LC Positive | 0-3 | No | 26 | 2.08 | 1.2 | 1 | 3 | 3 | 2 | 0 | 3 |
|  | PROQ3: LN Negative | 0-15 | No | 26 | 7.31 | 4.95 | 3 | 6.5 | 12 | 9 | 0 | 15 |
|  | PROQ3: LN Positive | 0-3 | No | 26 | 1.73 | 1.28 | 0 | 2 | 3 | 3 | 0 | 3 |
|  | PROQ3: LD Negative | 0-15 | No | 26 | 8.35 | 3.97 | 6 | 9 | 11 | 5 | 0 | 15 |
|  | PROQ3: LD Positive | 0-3 | No | 26 | 1.54 | 1.27 | 0 | 1 | 3 | 3 | 0 | 3 |
|  | PROQ3: ND Negative | 0-15 | No | 26 | 10.1 | 4.22 | 7 | 10 | 15 | 8 | 1 | 15 |
|  | PROQ3: ND Positive | 0-3 | No | 26 | 2.12 | 0.99 | 1 | 2.5 | 3 | 2 | 0 | 3 |
|  | PROQ3: UD Negative | 0-15 | No | 26 | 6.19 | 3.97 | 3 | 6 | 8 | 5 | 0 | 15 |
|  | PROQ3: UD Positive | 0-3 | No | 26 | 1.81 | 1.27 | 1 | 2 | 3 | 2 | 0 | 3 |
|  | PROQ3: Total | 0-144 | Yes | 26 | 71.2 | 17.9 | 58 | 70.5 | 84 | 26 | 44 | 106 |
|  | PTS: Ideas of Reference | 16-80 | No | 26 | 45.1 | 17.2 | 32 | 50.5 | 55 | 23 | 16 | 76 |
|  | PTS: Ideas of Persecution | 16-80 | No | 26 | 44.8 | 16.8 | 29 | 46 | 59 | 30 | 16 | 70 |
|  | WSNS: Total | 0-40 | Yes | 26 | 26.8 | 8.4 | 19 | 28 | 32 | 13 | 6 | 39 |
|  | SOFAS: Total | 0-100 | Yes | 26 | 44.7 | 12.4 | 35 | 43 | 59 | 24 | 23 | 65 |
|  |  |  |  |  |  |  |  |  |  |  |  |  |
| **Time point & Group** | **Outcome** | **Score Possible Range** | **Normally Distributed** | **N** | **mean** | **sd** | **25^th^ centile** | **median** | **75^th^ centile** | **IQR** | **min** | **max** |
| T0 | HPSVQ: Voice Characteristics | 0-20 | Yes | 26 | 13.8 | 2.59 | 12 | 14 | 15 | 3 | 7 | 18 |
| SC | PSYRATS: Frequency | 0-12 | Yes | 26 | 7.69 | 2.28 | 6 | 8 | 10 | 4 | 3 | 10 |
|  | PSYRATS: Loudness | 0-4 | Yes | 26 | 2.15 | 0.97 | 2 | 2 | 2 | 0 | 1 | 4 |
|  | PSYRATS: Attribution | 0-8 | Yes | 26 | 4.5 | 1.61 | 3 | 4.5 | 6 | 3 | 2 | 8 |
|  | PSYRATS: Total | 0-44 | Yes | 26 | 28.2 | 6.82 | 24 | 29 | 32 | 8 | 11 | 41 |
|  | VIS: Negative Impact | 0-80 | No | 25 | 25.8 | 12.1 | 18 | 26 | 33 | 15 | 0 | 54 |
|  | VIS: Positive Impact | 0-80 | No | 24 | 23.2 | 20.6 | 4.5 | 19 | 40.5 | 36 | 0 | 65 |
|  | VIS: Living Well | 0-80 | Yes | 24 | 39 | 16.6 | 28 | 42.5 | 52.5 | 24.5 | 1 | 66 |
|  | VIS: Total | 0-240 | Yes | 24 | 87.1 | 40.8 | 66 | 81 | 120 | 54 | 1 | 151 |
|  | HADS: Depression | 0-21 | Yes | 26 | 8.38 | 3.38 | 6 | 8 | 10 | 4 | 2 | 17 |
|  | HADS: Anxiety | 0-21 | Yes | 26 | 11.6 | 4.37 | 8 | 11.5 | 15 | 7 | 4 | 21 |
|  | CHOICE-SF: Mean | 0-10 | Yes | 26 | 4.49 | 1.69 | 3.18 | 5 | 5.55 | 2.36 | 0 | 7.36 |
|  | CHOICE-SF: Goal Rating | 0-10 | Yes | 26 | 3.73 | 2.25 | 2 | 5 | 5 | 3 | 0 | 8 |
|  | BCSS: Negative Self | 0-24 | No | 26 | 9.19 | 6.6 | 4 | 7.5 | 14 | 10 | 1 | 24 |
|  | BCSS: Positive Self | 0-24 | No | 26 | 8.54 | 5.04 | 4 | 9 | 12 | 8 | 0 | 21 |
|  | VAY: Voice Dominance | 0-21 | No | 26 | 15.3 | 4.48 | 12 | 15 | 20 | 8 | 6 | 21 |
|  | VAY: Voice Intrusiveness | 0-15 | No | 26 | 9.65 | 4.44 | 6 | 12 | 13 | 7 | 0 | 15 |
|  | VAY: Hearer Dependence | 0-27 | No | 26 | 11 | 6.35 | 6 | 12 | 15 | 9 | 0 | 24 |
|  | VAY: Hearer Distance | 0-21 | No | 26 | 13.8 | 4.94 | 11 | 14 | 18 | 7 | 3 | 21 |
|  | BAVQ: Persecutory | 0-27 | Yes | 26 | 14.7 | 6.43 | 11 | 15.5 | 19 | 8 | 3 | 27 |
|  | BAVQ: Benevolence | 0-15 | No | 26 | 3.38 | 4.43 | 0 | 0.5 | 6 | 6 | 0 | 15 |
|  | PROQ3: UN Negative | 0-15 | No | 26 | 5.77 | 4.08 | 3 | 5 | 8 | 5 | 0 | 15 |
|  | PROQ3: UN Positive | 0-3 | No | 26 | 0.462 | 0.86 | 0 | 0 | 1 | 1 | 0 | 3 |
|  | PROQ3: UC Negative | 0-15 | No | 26 | 4.08 | 4.02 | 0 | 3 | 6 | 6 | 0 | 15 |
|  | PROQ3: UC Positive | 0-3 | No | 26 | 2.65 | 0.69 | 2 | 3 | 3 | 1 | 0 | 3 |
|  | PROQ3: NC Negative | 0-15 | No | 26 | 5.69 | 4.55 | 2 | 5 | 9 | 7 | 0 | 15 |
|  | PROQ3: NC Positive | 0-3 | No | 26 | 1.69 | 1.35 | 0 | 2 | 3 | 3 | 0 | 3 |
|  | PROQ3: LC Negative | 0-15 | No | 26 | 8.69 | 3.83 | 6 | 8.5 | 13 | 7 | 3 | 15 |
|  | PROQ3: LC Positive | 0-3 | No | 26 | 2.58 | 0.81 | 2 | 3 | 3 | 1 | 0 | 3 |
|  | PROQ3: LN Negative | 0-15 | No | 26 | 8.15 | 4.55 | 4 | 8.5 | 11 | 7 | 0 | 15 |
|  | PROQ3: LN Positive | 0-3 | No | 26 | 2.19 | 1.17 | 1 | 3 | 3 | 2 | 0 | 3 |
|  | PROQ3: LD Negative | 0-15 | No | 26 | 7.69 | 4.21 | 4 | 8 | 10 | 6 | 1 | 15 |
|  | PROQ3: LD Positive | 0-3 | No | 26 | 1.85 | 1.19 | 1 | 2 | 3 | 2 | 0 | 3 |
|  | PROQ3: ND Negative | 0-15 | No | 26 | 10 | 4.28 | 6 | 10.5 | 14 | 8 | 2 | 15 |
|  | PROQ3: ND Positive | 0-3 | No | 26 | 1.31 | 1.23 | 0 | 1 | 3 | 3 | 0 | 3 |
|  | PROQ3: UD Negative | 0-15 | No | 26 | 5.77 | 3.88 | 2 | 6 | 9 | 7 | 0 | 12 |
|  | PROQ3: UD Positive | 0-3 | No | 26 | 1.88 | 1.34 | 0 | 3 | 3 | 3 | 0 | 3 |
|  | PROQ3: Total | 0-144 | Yes | 26 | 70.5 | 20.6 | 51 | 68.5 | 89 | 38 | 34 | 108 |
|  | PTS: Ideas of Reference | 16-80 | No | 26 | 46.3 | 18.9 | 30 | 41 | 63 | 33 | 16 | 79 |
|  | PTS: Ideas of Persecution | 16-80 | No | 26 | 47 | 20.5 | 29 | 49.5 | 65 | 36 | 16 | 80 |
|  | WSNS: Total | 0-40 | Yes | 26 | 26.3 | 8.97 | 21 | 28 | 33 | 12 | 6 | 40 |
|  | SOFAS: Total | 0-100 | Yes | 26 | 43.6 | 13.2 | 32 | 45 | 51 | 19 | 20 | 68 |
|  |  |  |  |  |  |  |  |  |  |  |  |  |
|  |  |  |  |  |  |  |  |  |  |  |  |  |
|  |  |  |  |  |  |  |  |  |  |  |  |  |
| **Time point & Group** | **Outcome** | **Score Possible Range** | **Normally Distributed** | **N** | **mean** | **sd** | **25^th^ centile** | **median** | **75^th^ centile** | **IQR** | **min** | **max** |
| T1 | HPSVQ: Voice Characteristics | 0-20 | Yes | 23 | 11.6 | 4.29 | 10 | 13 | 14 | 4 | 0 | 16 |
| TAU | PSYRATS: Frequency | 0-12 | Yes | 23 | 6.3 | 2.62 | 5 | 7 | 8 | 3 | 0 | 10 |
|  | PSYRATS: Loudness | 0-4 | Yes | 23 | 2.43 | 1.2 | 2 | 2 | 3 | 1 | 0 | 4 |
|  | PSYRATS: Attribution | 0-8 | Yes | 23 | 4.96 | 2.25 | 4 | 5 | 7 | 3 | 0 | 8 |
|  | PSYRATS: Total | 0-44 | Yes | 23 | 27.3 | 9.36 | 27 | 29 | 34 | 7 | 1 | 36 |
|  | VIS: Negative Impact | 0-80 | No | 22 | 21.2 | 12.1 | 14 | 21.5 | 28 | 14 | 0 | 46 |
|  | VIS: Positive Impact | 0-80 | No | 22 | 7.45 | 12.1 | 0 | 2 | 7 | 7 | 0 | 39 |
|  | VIS: Living Well | 0-80 | Yes | 22 | 32.2 | 15.3 | 19 | 35 | 43 | 24 | 7 | 59 |
|  | VIS: Total | 0-240 | Yes | 22 | 60.9 | 27.1 | 48 | 60 | 74 | 26 | 7 | 126 |
|  | HADS: Depression | 0-21 | Yes | 23 | 11.1 | 4.89 | 8 | 10 | 15 | 7 | 1 | 18 |
|  | HADS: Anxiety | 0-21 | Yes | 23 | 13.1 | 4.1 | 9 | 13 | 16 | 7 | 5 | 20 |
|  | CHOICE-SF: Mean | 0-10 | Yes | 23 | 3.85 | 1.81 | 1.82 | 3.73 | 5.64 | 3.82 | 1.36 | 7.18 |
|  | CHOICE-SF: Goal Rating | 0-10 | Yes | 23 | 3.52 | 2.06 | 2 | 3 | 5 | 3 | 0 | 7 |
|  | BCSS: Negative Self | 0-24 | No | 23 | 11.8 | 5.67 | 7 | 12 | 16 | 9 | 2 | 23 |
|  | BCSS: Positive Self | 0-24 | No | 23 | 5.48 | 4.28 | 1 | 6 | 10 | 9 | 0 | 12 |
|  | VAY: Voice Dominance | 0-21 | No | 22 | 17.2 | 5.37 | 18 | 19 | 21 | 3 | 1 | 21 |
|  | VAY: Voice Intrusiveness | 0-15 | No | 23 | 8.96 | 3.98 | 6 | 9 | 12 | 6 | 3 | 15 |
|  | VAY: Hearer Dependence | 0-27 | No | 23 | 6.3 | 4.82 | 3 | 5 | 9 | 6 | 0 | 18 |
|  | VAY: Hearer Distance | 0-21 | No | 23 | 15.8 | 3.37 | 14 | 15 | 19 | 5 | 8 | 21 |
|  | BAVQ: Persecutory | 0-27 | Yes | 23 | 15 | 5.18 | 11 | 15 | 19 | 8 | 4 | 24 |
|  | BAVQ: Benevolence | 0-15 | No | 23 | 1.17 | 2.46 | 0 | 0 | 1 | 1 | 0 | 9 |
|  | PROQ3: UN Negative | 0-15 | No | 23 | 5.3 | 4.54 | 1 | 5 | 9 | 8 | 0 | 15 |
|  | PROQ3: UN Positive | 0-3 | No | 23 | 1.04 | 1.19 | 0 | 1 | 2 | 2 | 0 | 3 |
|  | PROQ3: UC Negative | 0-15 | No | 23 | 3.09 | 3.8 | 0 | 2 | 5 | 5 | 0 | 12 |
|  | PROQ3: UC Positive | 0-3 | No | 23 | 2.48 | 0.90 | 2 | 3 | 3 | 1 | 0 | 3 |
|  | PROQ3: NC Negative | 0-15 | No | 23 | 4.7 | 4.84 | 1 | 3 | 9 | 8 | 0 | 15 |
|  | PROQ3: NC Positive | 0-3 | No | 23 | 1.22 | 1.24 | 0 | 1 | 3 | 3 | 0 | 3 |
|  | PROQ3: LC Negative | 0-15 | No | 23 | 9.96 | 3.23 | 7 | 10 | 12 | 5 | 3 | 15 |
|  | PROQ3: LC Positive | 0-3 | No | 23 | 2.35 | 0.98 | 2 | 3 | 3 | 1 | 0 | 3 |
|  | PROQ3: LN Negative | 0-15 | No | 23 | 8.22 | 3.73 | 6 | 8 | 12 | 6 | 0 | 15 |
|  | PROQ3: LN Positive | 0-3 | No | 22 | 2.14 | 1.08 | 2 | 2.5 | 3 | 1 | 0 | 3 |
|  | PROQ3: LD Negative | 0-15 | No | 23 | 9.39 | 3.31 | 7 | 9 | 12 | 5 | 2 | 15 |
|  | PROQ3: LD Positive | 0-3 | No | 23 | 1.83 | 0.98 | 1 | 2 | 3 | 2 | 0 | 3 |
|  | PROQ3: ND Negative | 0-15 | No | 23 | 10.4 | 3.74 | 7 | 10 | 15 | 8 | 5 | 15 |
|  | PROQ3: ND Positive | 0-3 | No | 23 | 2.22 | 1.09 | 1 | 3 | 3 | 2 | 0 | 3 |
|  | PROQ3: UD Negative | 0-15 | No | 23 | 4 | 3.21 | 1 | 3 | 7 | 6 | 0 | 10 |
|  | PROQ3: UD Positive | 0-3 | No | 23 | 1.13 | 1.25 | 0 | 1 | 2 | 2 | 0 | 3 |
|  | PROQ3: Total | 0-144 | Yes | 22 | 70 | 17.8 | 58 | 66 | 74 | 16 | 47 | 116 |
|  | PTS: Ideas of Reference | 16-80 | No | 23 | 46.6 | 16.9 | 36 | 44 | 56 | 20 | 16 | 76 |
|  | PTS: Ideas of Persecution | 16-80 | No | 23 | 44 | 18.5 | 28 | 51 | 59 | 31 | 16 | 76 |
|  | WSNS: Total | 0-40 | Yes | 23 | 25.4 | 7.98 | 20 | 25 | 32 | 12 | 9 | 38 |
|  | SOFAS: Total | 0-100 | Yes | 23 | 42 | 7.89 | 35 | 40 | 50 | 15 | 30 | 60 |
|  |  |  |  |  |  |  |  |  |  |  |  |  |
|  |  |  |  |  |  |  |  |  |  |  |  |  |
| **Time point & Group** | **Clinical Outcome** | **Score Possible Range** | **Normally Distributed** | **N** | **mean** | **sd** | **25^th^ centile** | **median** | **75^th^ centile** | **IQR** | **min** | **max** |
| T1 | HPSVQ: Voice Characteristics | 0-20 | Yes | 22 | 10.6 | 4.49 | 10 | 11 | 13 | 3 | 0 | 18 |
| GiVE | PSYRATS: Frequency | 0-12 | Yes | 22 | 5.73 | 2.85 | 4 | 5 | 8 | 4 | 0 | 10 |
|  | PSYRATS: Loudness | 0-4 | Yes | 22 | 2.09 | 1.02 | 2 | 2 | 3 | 1 | 0 | 4 |
|  | PSYRATS: Attribution | 0-8 | Yes | 22 | 3.77 | 1.9 | 3 | 4 | 5 | 2 | 0 | 7 |
|  | PSYRATS: Total | 0-44 | Yes | 22 | 23.4 | 9.5 | 21 | 26 | 29 | 8 | 0 | 35 |
|  | VIS: Negative Impact | 0-80 | No | 20 | 30.1 | 17 | 18.5 | 26.5 | 39.5 | 21 | 4 | 73 |
|  | VIS: Positive Impact | 0-80 | No | 19 | 13.1 | 14 | 0 | 9 | 22 | 22 | 0 | 47 |
|  | VIS: Living Well | 0-80 | Yes | 18 | 41.1 | 12.2 | 32 | 43 | 48 | 16 | 17 | 67 |
|  | VIS: Total | 0-240 | Yes | 17 | 82.5 | 27 | 64 | 87 | 95 | 31 | 26 | 140 |
|  | HADS: Depression | 0-21 | Yes | 22 | 8.86 | 3.63 | 7 | 9.5 | 12 | 5 | 0 | 14 |
|  | HADS: Anxiety | 0-21 | Yes | 22 | 11.9 | 4.38 | 10 | 11.5 | 15 | 5 | 2 | 19 |
|  | CHOICE-SF: Mean | 0-10 | Yes | 22 | 5.63 | 2.13 | 4.09 | 5.36 | 7.18 | 3.09 | 1.27 | 9.27 |
|  | CHOICE-SF: Goal Rating | 0-10 | Yes | 22 | 6.59 | 2.56 | 5 | 7 | 9 | 4 | 2 | 10 |
|  | BCSS: Negative Self | 0-24 | No | 22 | 6 | 5.79 | 0 | 5 | 9 | 9 | 0 | 21 |
|  | BCSS: Positive Self | 0-24 | No | 22 | 11.1 | 7.36 | 4 | 11.5 | 17 | 13 | 0 | 24 |
|  | VAY: Voice Dominance | 0-21 | No | 22 | 12.9 | 4.94 | 9 | 13 | 16 | 7 | 4 | 21 |
|  | VAY: Voice Intrusiveness | 0-15 | No | 22 | 8.68 | 4.86 | 6 | 10 | 12 | 6 | 0 | 15 |
|  | VAY: Hearer Dependence | 0-27 | No | 22 | 6.68 | 5.29 | 3 | 6.5 | 10 | 7 | 0 | 16 |
|  | VAY: Hearer Distance | 0-21 | No | 22 | 14.7 | 4.57 | 13 | 14 | 18 | 5 | 5 | 21 |
|  | BAVQ: Persecutory | 0-27 | Yes | 22 | 11.8 | 6.38 | 8 | 12 | 17 | 9 | 0 | 22 |
|  | BAVQ: Benevolence | 0-15 | No | 22 | 1.77 | 3.37 | 0 | 0 | 1 | 1 | 0 | 10 |
|  | PROQ3: UN Negative | 0-15 | No | 22 | 4.5 | 4.07 | 1 | 3 | 8 | 7 | 0 | 15 |
|  | PROQ3: UN Positive | 0-3 | No | 22 | 1.27 | 1.12 | 0 | 1 | 2 | 2 | 0 | 3 |
|  | PROQ3: UC Negative | 0-15 | No | 22 | 3.68 | 4.52 | 0 | 1.5 | 9 | 9 | 0 | 12 |
|  | PROQ3: UC Positive | 0-3 | No | 22 | 2.55 | 0.8 | 2 | 3 | 3 | 1 | 1 | 3 |
|  | PROQ3: NC Negative | 0-15 | No | 22 | 4.86 | 3.91 | 2 | 3.5 | 7 | 5 | 0 | 15 |
|  | PROQ3: NC Positive | 0-3 | No | 22 | 1.73 | 1.32 | 0 | 2 | 3 | 3 | 0 | 3 |
|  | PROQ3: LC Negative | 0-15 | No | 22 | 7.27 | 4.86 | 3 | 6.5 | 12 | 9 | 0 | 15 |
|  | PROQ3: LC Positive | 0-3 | No | 22 | 2.32 | 1.13 | 2 | 3 | 3 | 1 | 0 | 3 |
|  | PROQ3: LN Negative | 0-15 | No | 22 | 6.82 | 4.59 | 3 | 6 | 12 | 9 | 0 | 15 |
|  | PROQ3: LN Positive | 0-3 | No | 22 | 2.23 | 1.02 | 2 | 3 | 3 | 1 | 0 | 3 |
|  | PROQ3: LD Negative | 0-15 | No | 22 | 8.73 | 4.37 | 5 | 9 | 12 | 7 | 1 | 15 |
|  | PROQ3: LD Positive | 0-3 | No | 22 | 1.77 | 1.27 | 1 | 2 | 3 | 2 | 0 | 3 |
|  | PROQ3: ND Negative | 0-15 | No | 22 | 9.77 | 3.72 | 7 | 9 | 12 | 5 | 3 | 15 |
|  | PROQ3: ND Positive | 0-3 | No | 22 | 2 | 1.02 | 1 | 2 | 3 | 2 | 0 | 3 |
|  | PROQ3: UD Negative | 0-15 | No | 22 | 5.77 | 3.99 | 3 | 4.5 | 9 | 6 | 0 | 12 |
|  | PROQ3: UD Positive | 0-3 | No | 22 | 1.86 | 1.25 | 1 | 2.5 | 3 | 2 | 0 | 3 |
|  | PROQ3: Total | 0-144 | Yes | 22 | 67.1 | 21.9 | 48 | 63.5 | 80 | 32 | 37 | 120 |
|  | PTS: Ideas of Reference | 16-80 | No | 22 | 41.4 | 16.5 | 27 | 40 | 57 | 30 | 18 | 74 |
|  | PTS: Ideas of Persecution | 16-80 | No | 22 | 41.4 | 20.1 | 24 | 39 | 54 | 30 | 16 | 80 |
|  | WSNS: Total | 0-40 | Yes | 22 | 22.9 | 8.66 | 17 | 24 | 28 | 11 | 6 | 40 |
|  | SOFAS: Total | 0-100 | Yes | 22 | 50.7 | 15.2 | 40 | 45 | 65 | 25 | 31 | 81 |
|  |  |  |  |  |  |  |  |  |  |  |  |  |
| **Time point & Group** | **Outcome** | **Score Possible Range** | **Normally Distributed** | **N** | **mean** | **sd** | **25^th^ centile** | **median** | **75^th^ centile** | **IQR** | **min** | **max** |
| T1 | HPSVQ: Voice Characteristics | 0-20 | Yes | 23 | 13.4 | 2.73 | 12 | 14 | 16 | 4 | 7 | 17 |
| SC | PSYRATS: Frequency | 0-12 | Yes | 23 | 7.39 | 2.1 | 6 | 8 | 9 | 3 | 4 | 10 |
|  | PSYRATS: Loudness | 0-4 | Yes | 23 | 2.3 | 1.02 | 2 | 2 | 3 | 1 | 1 | 4 |
|  | PSYRATS: Attribution | 0-8 | Yes | 23 | 4.57 | 1.93 | 3 | 5 | 6 | 3 | 2 | 8 |
|  | PSYRATS: Total | 0-44 | Yes | 22 | 28.2 | 6.34 | 25 | 28.5 | 32 | 7 | 15 | 40 |
|  | VIS: Negative Impact | 0-80 | No | 22 | 27.8 | 20.5 | 12 | 25.5 | 43 | 31 | 0 | 70 |
|  | VIS: Positive Impact | 0-80 | No | 21 | 19.2 | 20.9 | 0 | 10 | 34 | 34 | 0 | 61 |
|  | VIS: Living Well | 0-80 | Yes | 21 | 36.1 | 19.8 | 23 | 37 | 47 | 24 | 0 | 72 |
|  | VIS: Total | 0-240 | Yes | 20 | 83.3 | 48 | 46 | 83.5 | 120 | 74 | 0 | 163 |
|  | HADS: Depression | 0-21 | Yes | 23 | 8.57 | 4.65 | 5 | 8 | 11 | 6 | 0 | 18 |
|  | HADS: Anxiety | 0-21 | Yes | 23 | 11.6 | 3.96 | 9 | 11 | 15 | 6 | 3 | 18 |
|  | CHOICE-SF: Mean | 0-10 | Yes | 23 | 5.19 | 2.23 | 3.27 | 5.82 | 6.91 | 3.64 | 0 | 7.73 |
|  | CHOICE-SF: Goal Rating | 0-10 | Yes | 23 | 5.3 | 3.23 | 3 | 6 | 8 | 5 | 0 | 10 |
|  | BCSS: Negative Self | 0-24 | No | 23 | 6.3 | 6.77 | 1 | 4 | 9 | 8 | 0 | 24 |
|  | BCSS: Positive Self | 0-24 | No | 23 | 9.3 | 6.26 | 4 | 10 | 14 | 10 | 0 | 23 |
|  | VAY: Voice Dominance | 0-21 | No | 23 | 13.4 | 6.77 | 8 | 15 | 19 | 11 | 0 | 21 |
|  | VAY: Voice Intrusiveness | 0-15 | No | 23 | 9.7 | 4.44 | 9 | 11 | 12 | 3 | 0 | 15 |
|  | VAY: Hearer Dependence | 0-27 | No | 23 | 7.78 | 6.69 | 3 | 6 | 13 | 10 | 0 | 24 |
|  | VAY: Hearer Distance | 0-21 | No | 23 | 16.2 | 3.93 | 13 | 17 | 18 | 5 | 7 | 21 |
|  | BAVQ: Persecutory | 0-27 | Yes | 23 | 13.4 | 8.02 | 6 | 14 | 20 | 14 | 0 | 27 |
|  | BAVQ: Benevolence | 0-15 | No | 23 | 3.48 | 4.96 | 0 | 1 | 5 | 5 | 0 | 15 |
|  | PROQ3: UN Negative | 0-15 | No | 23 | 4.78 | 3.74 | 2 | 3 | 8 | 6 | 0 | 13 |
|  | PROQ3: UN Positive | 0-3 | No | 23 | 0.70 | 1.02 | 0 | 0 | 1 | 1 | 0 | 3 |
|  | PROQ3: UC Negative | 0-15 | No | 23 | 4 | 3.19 | 1 | 3 | 7 | 6 | 0 | 11 |
|  | PROQ3: UC Positive | 0-3 | No | 23 | 2.74 | 0.752 | 3 | 3 | 3 | 0 | 0 | 3 |
|  | PROQ3: NC Negative | 0-15 | No | 23 | 6.26 | 3.79 | 4 | 7 | 9 | 5 | 0 | 12 |
|  | PROQ3: NC Positive | 0-3 | No | 23 | 1.57 | 1.34 | 0 | 1 | 3 | 3 | 0 | 3 |
|  | PROQ3: LC Negative | 0-15 | No | 23 | 8.13 | 4.19 | 5 | 9 | 11 | 6 | 1 | 15 |
|  | PROQ3: LC Positive | 0-3 | No | 23 | 2.26 | 1.21 | 1 | 3 | 3 | 2 | 0 | 3 |
|  | PROQ3: LN Negative | 0-15 | No | 23 | 7.96 | 3.76 | 6 | 9 | 10 | 4 | 1 | 15 |
|  | PROQ3: LN Positive | 0-3 | No | 23 | 2.3 | 1.02 | 2 | 3 | 3 | 1 | 0 | 3 |
|  | PROQ3: LD Negative | 0-15 | No | 23 | 8.7 | 3.84 | 6 | 10 | 11 | 5 | 0 | 15 |
|  | PROQ3: LD Positive | 0-3 | No | 23 | 1.26 | 0.96 | 1 | 1 | 2 | 1 | 0 | 3 |
|  | PROQ3: ND Negative | 0-15 | No | 23 | 8.35 | 3.68 | 6 | 9 | 12 | 6 | 0 | 14 |
|  | PROQ3: ND Positive | 0-3 | No | 23 | 1.61 | 1.23 | 1 | 1 | 3 | 2 | 0 | 3 |
|  | PROQ3: UD Negative | 0-15 | No | 23 | 5.48 | 3.42 | 3 | 6 | 8 | 5 | 0 | 12 |
|  | PROQ3: UD Positive | 0-3 | No | 23 | 1.96 | 1.22 | 1 | 3 | 3 | 2 | 0 | 3 |
|  | PROQ3: Total | 0-144 | Yes | 23 | 68 | 16.4 | 59 | 68 | 81 | 22 | 33 | 97 |
|  | PTS: Ideas of Reference | 16-80 | No | 23 | 40.3 | 17.3 | 24 | 41 | 50 | 26 | 16 | 76 |
|  | PTS: Ideas of persecution | 16-80 | No | 23 | 41.1 | 21.2 | 21 | 36 | 54 | 33 | 16 | 80 |
|  | WSNS: Total | 0-40 | Yes | 23 | 23.3 | 9.79 | 16 | 25 | 30 | 14 | 4 | 39 |
|  | SOFAS: Total | 0-100 | Yes | 23 | 50.5 | 14.9 | 35 | 50 | 62 | 27 | 31 | 80 |
|  |  |  |  |  |  |  |  |  |  |  |  |  |
|  |  |  |  |  |  |  |  |  |  |  |  |  |
|  |  |  |  |  |  |  |  |  |  |  |  |  |
|  |  |  |  |  |  |  |  |  |  |  |  |  |
| **Time point & Group** | **Outcome** | **Score Possible Range** | **Normally Distributed** | **N** | **mean** | **sd** | **25^th^ centile** | **median** | **75^th^ centile** | **IQR** | **min** | **max** |
| T2 | HPSVQ: Voice Characteristics | 0-20 | Yes | 14 | 12.3 | 3.22 | 10 | 12 | 15 | 5 | 7 | 17 |
| TAU | PSYRATS: Frequency | 0-12 | Yes | 15 | 6.67 | 1.91 | 5 | 6 | 7 | 2 | 4 | 10 |
|  | PSYRATS: Loudness | 0-4 | Yes | 15 | 2.73 | 0.88 | 2 | 3 | 3 | 1 | 1 | 4 |
|  | PSYRATS: Attribution | 0-8 | Yes | 15 | 5 | 2 | 3 | 5 | 6 | 3 | 2 | 8 |
|  | PSYRATS: Total | 0-44 | Yes | 15 | 28.7 | 5.2 | 25 | 31 | 33 | 8 | 17 | 36 |
|  | VIS: Negative Impact | 0-80 | No | 15 | 21.5 | 10.5 | 14 | 21 | 27 | 13 | 5 | 42 |
|  | VIS: Positive Impact | 0-80 | No | 15 | 6.07 | 10.4 | 0 | 2 | 6 | 6 | 0 | 39 |
|  | VIS: Living Well | 0-80 | Yes | 15 | 33.1 | 13 | 25 | 32 | 41 | 16 | 12 | 55 |
|  | VIS: Total | 0-240 | Yes | 15 | 60.7 | 20.4 | 48 | 58 | 79 | 31 | 26 | 100 |
|  | HADS: Depression | 0-21 | Yes | 15 | 10.5 | 3.8 | 7 | 11 | 13 | 6 | 4 | 17 |
|  | HADS: Anxiety | 0-21 | Yes | 15 | 11.9 | 4.37 | 8 | 13 | 16 | 8 | 5 | 18 |
|  | CHOICE-SF: Mean | 0-10 | Yes | 15 | 4.15 | 1.65 | 2.45 | 4.45 | 5.64 | 3.18 | 1.36 | 6.55 |
|  | CHOICE-SF: Goal Rating | 0-10 | Yes | 14 | 3.43 | 2.53 | 2 | 3 | 6 | 4 | 0 | 8 |
|  | BCSS: Negative Self | 0-24 | No | 15 | 8.13 | 5.79 | 3 | 6 | 12 | 9 | 2 | 20 |
|  | BCSS: Positive Self | 0-24 | No | 15 | 5.33 | 4.58 | 1 | 6 | 10 | 9 | 0 | 14 |
|  | VAY: Voice Dominance | 0-21 | No | 15 | 16.9 | 5.44 | 12 | 19 | 21 | 9 | 3 | 21 |
|  | VAY: Voice Intrusiveness | 0-15 | No | 15 | 7.87 | 4.97 | 4 | 6 | 15 | 11 | 1 | 15 |
|  | VAY: Hearer Dependence | 0-27 | No | 15 | 3.67 | 5.01 | 0 | 3 | 4 | 4 | 0 | 20 |
|  | VAY: Hearer Distance | 0-21 | No | 15 | 15.1 | 3.39 | 13 | 15 | 18 | 5 | 9 | 20 |
|  | BAVQ: Persecutory | 0-27 | Yes | 15 | 14.5 | 4.84 | 12 | 14 | 18 | 6 | 7 | 22 |
|  | BAVQ: Benevolence | 0-15 | No | 15 | 1.07 | 1.87 | 0 | 0 | 2 | 2 | 0 | 6 |
|  | PROQ3: UN Negative | 0-15 | No | 15 | 5.2 | 4.16 | 1 | 4 | 9 | 8 | 0 | 12 |
|  | PROQ3: UN Positive | 0-3 | No | 15 | 0.87 | 1.25 | 0 | 0 | 2 | 2 | 0 | 3 |
|  | PROQ3: UC Negative | 0-15 | No | 15 | 3.4 | 4.42 | 0 | 1 | 7 | 7 | 0 | 12 |
|  | PROQ3: UC Positive | 0-3 | No | 15 | 2.27 | 1.03 | 1 | 3 | 3 | 2 | 0 | 3 |
|  | PROQ3: NC Negative | 0-15 | No | 15 | 6.07 | 5.4 | 2 | 4 | 12 | 10 | 0 | 15 |
|  | PROQ3: NC Positive | 0-3 | No | 15 | 1.2 | 1.37 | 0 | 0 | 3 | 3 | 0 | 3 |
|  | PROQ3: LC Negative | 0-15 | No | 15 | 9.07 | 3.58 | 6 | 9 | 12 | 6 | 3 | 15 |
|  | PROQ3: LC Positive | 0-3 | No | 15 | 2.67 | 0.62 | 2 | 3 | 3 | 1 | 1 | 3 |
|  | PROQ3: LN Negative | 0-15 | No | 15 | 7.13 | 5.15 | 1 | 7 | 11 | 10 | 0 | 15 |
|  | PROQ3: LN Positive | 0-3 | No | 15 | 2.13 | 0.99 | 1 | 2 | 3 | 2 | 0 | 3 |
|  | PROQ3: LD Negative | 0-15 | No | 15 | 9.07 | 4.22 | 6 | 8 | 13 | 7 | 3 | 15 |
|  | PROQ3: LD Positive | 0-3 | No | 15 | 1.47 | 1.19 | 0 | 1 | 3 | 3 | 0 | 3 |
|  | PROQ3: ND Negative | 0-15 | No | 15 | 9.87 | 2.88 | 7 | 10 | 12 | 5 | 5 | 15 |
|  | PROQ3: ND Positive | 0-3 | No | 15 | 2.07 | 1.22 | 1 | 3 | 3 | 2 | 0 | 3 |
|  | PROQ3: UD Negative | 0-15 | No | 15 | 4 | 3.8 | 0 | 3 | 8 | 8 | 0 | 10 |
|  | PROQ3: UD Positive | 0-3 | No | 15 | 1.2 | 1.26 | 0 | 1 | 3 | 3 | 0 | 3 |
|  | PROQ3: Total | 0-144 | Yes | 15 | 67.7 | 23.6 | 46 | 64 | 97 | 51 | 39 | 105 |
|  | PTS: Ideas of Reference | 16-80 | No | 15 | 36.5 | 15.1 | 22 | 38 | 44 | 22 | 16 | 70 |
|  | PTS: Ideas of Persecution | 16-80 | No | 15 | 35.8 | 17.9 | 20 | 37 | 44 | 24 | 16 | 78 |
|  | WSNS: Total | 0-40 | Yes | 16 | 26.3 | 6.88 | 21 | 25.5 | 29.5 | 8.5 | 16 | 39 |
|  | SOFAS: Total | 0-100 | Yes | 16 | 43.7 | 10.8 | 34 | 42 | 52 | 18 | 28 | 63 |
|  |  |  |  |  |  |  |  |  |  |  |  |  |
|  |  |  |  |  |  |  |  |  |  |  |  |  |
| **Time point & Group** | **Outcome** | **Score Possible Range** | **Normally Distributed** | **N** | **mean** | **sd** | **25^th^ centile** | **median** | **75^th^ centile** | **IQR** | **min** | **max** |
| T2 | HPSVQ: Voice Characteristics | 0-20 | Yes | 12 | 12.4 | 4.81 | 10.5 | 13 | 15.5 | 5 | 0 | 18 |
| GiVE | PSYRATS: Frequency | 0-12 | Yes | 12 | 7.33 | 3.17 | 5.5 | 8.5 | 9 | 3.5 | 0 | 11 |
|  | PSYRATS: Loudness | 0-4 | Yes | 12 | 2.33 | 1.15 | 2 | 2 | 3 | 1 | 0 | 4 |
|  | PSYRATS: Attribution | 0-8 | Yes | 12 | 4.17 | 2.44 | 2.5 | 3.5 | 6.5 | 4 | 0 | 8 |
|  | PSYRATS: Total | 0-44 | Yes | 12 | 26.8 | 9.97 | 24 | 30 | 33 | 9 | 0 | 38 |
|  | VIS: Negative Impact | 0-80 | No | 12 | 27.7 | 23.2 | 9 | 24 | 44.5 | 35.5 | 0 | 80 |
|  | VIS: Positive Impact | 0-80 | No | 12 | 8.17 | 11.9 | 0 | 0.5 | 19.5 | 19.5 | 0 | 34 |
|  | VIS: Living Well | 0-80 | Yes | 12 | 34.8 | 18.7 | 23 | 34.5 | 48 | 25 | 0 | 64 |
|  | VIS: Total | 0-240 | Yes | 12 | 70.6 | 26.7 | 42 | 77.5 | 89.5 | 47.5 | 34 | 110 |
|  | HADS: Depression | 0-21 | Yes | 12 | 10 | 5.03 | 9.5 | 11 | 12.5 | 3 | 0 | 18 |
|  | HADS: Anxiety | 0-21 | Yes | 12 | 11.3 | 4.21 | 8 | 11 | 15 | 7 | 6 | 18 |
|  | CHOICE-SF: Mean | 0-10 | Yes | 12 | 4.76 | 2.53 | 3.32 | 4.45 | 6.23 | 2.91 | 0 | 10 |
|  | CHOICE-SF: Goal Rating | 0-10 | Yes | 12 | 5.83 | 3.3 | 3 | 6.5 | 8.5 | 5.5 | 0 | 10 |
|  | BCSS: Negative Self | 0-24 | No | 12 | 6.5 | 6.71 | 0.5 | 5.5 | 11 | 10.5 | 0 | 22 |
|  | BCSS: Positive Self | 0-24 | No | 12 | 9.5 | 7.57 | 5 | 9.5 | 11 | 6 | 0 | 24 |
|  | VAY: Voice Dominance | 0-21 | No | 12 | 13.6 | 6.26 | 8 | 13.5 | 19.5 | 11.5 | 3 | 21 |
|  | VAY: Voice Intrusiveness | 0-15 | No | 11 | 8.09 | 5.41 | 3 | 9 | 13 | 10 | 0 | 15 |
|  | VAY: Hearer Dependence | 0-27 | No | 12 | 6.42 | 6.14 | 1 | 4.5 | 12 | 11 | 0 | 15 |
|  | VAY: Hearer Distance | 0-21 | No | 12 | 14.8 | 3.38 | 14.5 | 15 | 17 | 2.5 | 6 | 19 |
|  | BAVQ: Persecutory | 0-27 | Yes | 12 | 12.3 | 8.38 | 6 | 10.5 | 21 | 15 | 0 | 23 |
|  | BAVQ: Benevolence | 0-15 | No | 12 | 1.5 | 2.07 | 0 | 0 | 3 | 3 | 0 | 6 |
|  | PROQ3: UN Negative | 0-15 | No | 12 | 5.42 | 5.02 | 1 | 4.5 | 8.5 | 7.5 | 0 | 15 |
|  | PROQ3: UN Positive | 0-3 | No | 12 | 1.17 | 1.19 | 0 | 1 | 2 | 2 | 0 | 3 |
|  | PROQ3: UC Negative | 0-15 | No | 12 | 4.75 | 5.12 | 0 | 3.5 | 8.5 | 8.5 | 0 | 15 |
|  | PROQ3: UC Positive | 0-3 | No | 12 | 2.33 | 1.07 | 1.5 | 3 | 3 | 1.5 | 0 | 3 |
|  | PROQ3: NC Negative | 0-15 | No | 12 | 5.17 | 4.93 | 1 | 4 | 8 | 7 | 0 | 15 |
|  | PROQ3: NC Positive | 0-3 | No | 12 | 1.17 | 1.34 | 0 | 0.5 | 2.5 | 2.5 | 0 | 3 |
|  | PROQ3: LC Negative | 0-15 | No | 12 | 7.17 | 3.56 | 5.5 | 7.5 | 9.5 | 4 | 1 | 14 |
|  | PROQ3: LC Positive | 0-3 | No | 12 | 2 | 1.04 | 1 | 2 | 3 | 2 | 0 | 3 |
|  | PROQ3: LN Negative | 0-15 | No | 12 | 6.33 | 4.83 | 2 | 6.5 | 10 | 8 | 0 | 15 |
|  | PROQ3: LN Positive | 0-3 | No | 12 | 2.08 | 1.08 | 1 | 2.5 | 3 | 2 | 0 | 3 |
|  | PROQ3: LD Negative | 0-15 | No | 12 | 7.75 | 3.65 | 4.5 | 8 | 10.5 | 6 | 2 | 13 |
|  | PROQ3: LD Positive | 0-3 | No | 12 | 1.42 | 1.31 | 0 | 1 | 3 | 3 | 0 | 3 |
|  | PROQ3: ND Negative | 0-15 | No | 12 | 9.42 | 4.21 | 6.5 | 10.5 | 11.5 | 5 | 1 | 15 |
|  | PROQ3: ND Positive | 0-3 | No | 12 | 2.25 | 0.87 | 1.5 | 2.5 | 3 | 1.5 | 1 | 3 |
|  | PROQ3: UD Negative | 0-15 | No | 12 | 7.58 | 4.87 | 5 | 6 | 11.5 | 6.5 | 0 | 15 |
|  | PROQ3: UD Positive | 0-3 | No | 12 | 1.58 | 1.16 | 1 | 1 | 3 | 2 | 0 | 3 |
|  | PROQ3: Total | 0-144 | Yes | 12 | 67.6 | 17.6 | 54 | 66 | 79.5 | 25.5 | 41 | 99 |
|  | PTS: Ideas of Reference | 16-80 | No | 12 | 41.4 | 18.1 | 25 | 40 | 55 | 30 | 18 | 72 |
|  | PTS: Ideas of Persecution | 16-80 | No | 12 | 42.6 | 22.9 | 19.5 | 41 | 64 | 44.5 | 16 | 76 |
|  | WSNS: Total | 0-40 | Yes | 12 | 19.8 | 11.2 | 11.5 | 21.5 | 26.5 | 15 | 0 | 40 |
|  | SOFAS: Total | 0-100 | Yes | 12 | 50.3 | 16.5 | 35 | 49 | 65.5 | 30.5 | 30 | 75 |
|  |  |  |  |  |  |  |  |  |  |  |  |  |
|  |  |  |  |  |  |  |  |  |  |  |  |  |
| **Time point & Group** | **Outcome** | **Score Possible Range** | **Normally Distributed** | **N** | **mean** | **sd** | **25^th^ centile** | **median** | **75^th^ centile** | **IQR** | **min** | **max** |
| T2 | HPSVQ: Voice Characteristics | 0-20 | Yes | 17 | 12.5 | 3.18 | 11 | 12 | 14 | 3 | 5 | 17 |
| SC | PSYRATS: Frequency | 0-12 | Yes | 16 | 7.19 | 2.32 | 5 | 8 | 9 | 4 | 3 | 10 |
|  | PSYRATS: Loudness | 0-4 | Yes | 16 | 2 | 1.15 | 1 | 2 | 2.5 | 1.5 | 1 | 4 |
|  | PSYRATS: Attribution | 0-8 | Yes | 17 | 4.65 | 1.87 | 3 | 5 | 5 | 2 | 2 | 8 |
|  | PSYRATS: Total | 0-44 | Yes | 14 | 25.4 | 6.07 | 20 | 25.5 | 29 | 9 | 17 | 37 |
|  | VIS: Negative Impact | 0-80 | No | 16 | 34.9 | 21.7 | 17.5 | 34.5 | 54 | 36.5 | 0 | 68 |
|  | VIS: Positive Impact | 0-80 | No | 15 | 24.7 | 22.9 | 2 | 19 | 45 | 43 | 0 | 70 |
|  | VIS: Living Well | 0-80 | Yes | 16 | 46.9 | 18.3 | 36 | 48 | 62.5 | 26.5 | 2 | 71 |
|  | VIS: Total | 0-240 | Yes | 15 | 106 | 50.4 | 64 | 113 | 149 | 85 | 4 | 184 |
|  | HADS: Depression | 0-21 | Yes | 16 | 7.38 | 4.36 | 4 | 7.5 | 10 | 6 | 1 | 17 |
|  | HADS: Anxiety | 0-21 | Yes | 16 | 10 | 5.85 | 4.5 | 10.5 | 14 | 9.5 | 1 | 20 |
|  | CHOICE-SF: Mean | 0-10 | Yes | 16 | 5.59 | 2.68 | 3.77 | 5.41 | 8.05 | 4.28 | 0 | 9 |
|  | CHOICE-SF: Goal Rating | 0-10 | Yes | 16 | 5.94 | 2.98 | 4.5 | 7 | 8 | 3.5 | 0 | 10 |
|  | BCSS: Negative Self | 0-24 | No | 15 | 7.73 | 6.72 | 1 | 8 | 13 | 12 | 0 | 20 |
|  | BCSS: Positive Self | 0-24 | No | 14 | 9.07 | 6.4 | 4 | 8 | 14 | 10 | 0 | 19 |
|  | VAY: Voice Dominance | 0-21 | No | 15 | 12 | 6.51 | 8 | 12 | 18 | 10 | 0 | 21 |
|  | VAY: Voice Intrusiveness | 0-15 | No | 15 | 8.4 | 4.58 | 5 | 7 | 12 | 7 | 2 | 15 |
|  | VAY: Hearer Dependence | 0-27 | No | 15 | 8.93 | 7.22 | 1 | 11 | 13 | 12 | 0 | 21 |
|  | VAY: Hearer Distance | 0-21 | No | 15 | 13.7 | 5.15 | 8 | 15 | 18 | 10 | 6 | 21 |
|  | BAVQ: Persecutory | 0-27 | Yes | 15 | 13.6 | 6.99 | 8 | 15 | 19 | 11 | 0 | 24 |
|  | BAVQ: Benevolence | 0-15 | No | 15 | 3.47 | 4.81 | 0 | 2 | 8 | 8 | 0 | 13 |
|  | PROQ3: UN Negative | 0-15 | No | 15 | 5.13 | 3.96 | 2 | 4 | 6 | 4 | 0 | 15 |
|  | PROQ3: UN Positive | 0-3 | No | 15 | 0.6 | 1.06 | 0 | 0 | 1 | 1 | 0 | 3 |
|  | PROQ3: UC Negative | 0-15 | No | 15 | 4.47 | 4.84 | 0 | 3 | 8 | 8 | 0 | 15 |
|  | PROQ3: UC Positive | 0-3 | No | 15 | 2.47 | 0.99 | 2 | 3 | 3 | 1 | 0 | 3 |
|  | PROQ3: NC Negative | 0-15 | No | 15 | 5.8 | 3.88 | 3 | 6 | 9 | 6 | 0 | 12 |
|  | PROQ3: NC Positive | 0-3 | No | 15 | 1.27 | 1.49 | 0 | 0 | 3 | 3 | 0 | 3 |
|  | PROQ3: LC Negative | 0-15 | No | 15 | 6.6 | 3.42 | 4 | 6 | 9 | 5 | 1 | 13 |
|  | PROQ3: LC Positive | 0-3 | No | 15 | 2.47 | 1.13 | 3 | 3 | 3 | 0 | 0 | 3 |
|  | PROQ3: LN Negative | 0-15 | No | 15 | 8.13 | 3.94 | 6 | 7 | 12 | 6 | 1 | 15 |
|  | PROQ3: LN Positive | 0-3 | No | 15 | 2.47 | 0.99 | 2 | 3 | 3 | 1 | 0 | 3 |
|  | PROQ3: LD Negative | 0-15 | No | 15 | 8.6 | 3.7 | 7 | 10 | 11 | 4 | 1 | 13 |
|  | PROQ3: LD Positive | 0-3 | No | 15 | 1.67 | 1.23 | 1 | 1 | 3 | 2 | 0 | 3 |
|  | PROQ3: ND Negative | 0-15 | No | 15 | 9.8 | 4.18 | 6 | 10 | 13 | 7 | 3 | 15 |
|  | PROQ3: ND Positive | 0-3 | No | 15 | 1.73 | 1.33 | 0 | 2 | 3 | 3 | 0 | 3 |
|  | PROQ3: UD Negative | 0-15 | No | 15 | 5 | 4.54 | 1 | 4 | 7 | 6 | 0 | 15 |
|  | PROQ3: UD Positive | 0-3 | No | 15 | 2 | 1.25 | 1 | 3 | 3 | 2 | 0 | 3 |
|  | PROQ3: Total | 0-144 | Yes | 15 | 68.2 | 20.3 | 52 | 67 | 83 | 31 | 30 | 101 |
|  | PTS: Ideas of Reference | 16-80 | No | 15 | 37.4 | 17.9 | 25 | 35 | 44 | 19 | 16 | 80 |
|  | PTS: Ideas of Persecution | 16-80 | No | 15 | 34.4 | 21.7 | 19 | 28 | 41 | 22 | 16 | 80 |
|  | WSNS: Total | 0-40 | Yes | 17 | 23.4 | 11.2 | 16 | 27 | 31 | 15 | 2 | 40 |
|  | SOFAS: Total | 0-100 | Yes | 17 | 54.5 | 17.1 | 41 | 50 | 70 | 29 | 30 | 81 |

Notes: N=count; sd=standard deviation; IQR=Interquartile Range; min=minimum value; max= maximum value; HPSVQ**=**Hamilton Program for Schizophrenia Voices Questionnaire; PSYRATS=Psychotic Symptoms Rating Scales; VIS=Voice Impact Scale; HADS=Hospital Anxiety and Depression Scale; CHOICE-SF=CHoice of Outcome In Cbt for psychosEs-Short Form ; BCSS=Brief Core Schema Scale; VAY = Voices And You; BAVQ=Beliefs About Voices Questionnaire; PROQ=Persons Relating to Others Questionnaire (UN=Upper Neutral, UC=Upper Close, NC, Neutral Close, LC, Lower Close, LN, Lower Neutral, LD, Lower Distant, ND=Neutral Distant, UD=Upper Distant) PTS=Paranoid Thoughts Scale; WSAS=Work and Social Adjustment Scale**;** SOFAS**=**Social and Occupational Functioning Scale; GiVE=Guided self-help CBT intervention for voices; TAU= Treatment as Usual; SC=Supportive Counselling

**Supplementary Table 2: Effect estimates for Primary & Secondary Clinical Outcomes by time point**

|  |  | **T1** | |  |  | |  | |  | |  | |  | |  | |  | | **T2** | |  |  |  |  |  |  |  |
| --- | --- | --- | --- | --- | --- | --- | --- | --- | --- | --- | --- | --- | --- | --- | --- | --- | --- | --- | --- | --- | --- | --- | --- | --- | --- | --- | --- |
| **Clinical Outcome** | **Pairwise Comparison** | **Effect**  **Estimate** | | **SE** | **95% LCL** | | **95% UCL** | | **75%**  **LCL** | | **75%**  **UCL** | | **SD** | | **Cohen's d** | |  | | **Effect**  **Estimate** | | **SE** | **95%**  **LCL** | **95% UCL** | **75% LCL** | **75%UCL** | **SD** | **Cohen's d** |
| HPSVQ: Voice Impact | GiVE vs TAU | -1.75 | 1.13 | | | -3.96 | | 0.46 | | -3.04 | | -0.45 | | 2.46 | | -0.71 | |  | | -0.37 | 1.37 | -3.06 | 2.32 | -1.95 | 1.21 | 2.46 | -0.15 |
|  | SC vs TAU | 0.08 | 1.11 | | | -2.09 | | 2.25 | | -1.19 | | 1.36 | | 2.46 | | 0.03 | |  | | -1.01 | 1.28 | -3.51 | 1.50 | -2.48 | 0.47 | 2.46 | -0.41 |
|  | SC vs GiVE | 1.83 | 1.12 | | | -0.36 | | 4.02 | | 0.55 | | 3.11 | | 2.46 | | 0.75 | |  | | -0.64 | 1.33 | -3.24 | 1.96 | -2.16 | 0.89 | 2.46 | -0.26 |
| HPSVQ: Voice Characteristics | GiVE vs TAU | -0.35 | 0.92 | | | -2.15 | | 1.45 | | -1.40 | | 0.71 | | 2.61 | | -0.13 | |  | | -0.42 | 1.12 | -2.62 | 1.78 | -1.71 | 0.87 | 2.61 | -0.16 |
|  | SC vs TAU | 1.76 | 0.90 | | | 0.00 | | 3.53 | | 0.73 | | 2.80 | | 2.61 | | 0.68 | |  | | -0.20 | 1.04 | -2.25 | 1.84 | -1.40 | 1.00 | 2.61 | -0.08 |
|  | SC vs GiVE | 2.11 | 0.91 | | | 0.32 | | 3.90 | | 1.06 | | 3.16 | | 2.61 | | 0.81 | |  | | 0.22 | 1.08 | -1.91 | 2.34 | -1.03 | 1.46 | 2.61 | 0.08 |
| PSYRATS: Distress | GiVE vs TAU | -1.63 | 1.19 | | | -3.97 | | 0.72 | | -3.00 | | -0.25 | | 3.30 | | -0.49 | |  | | -1.66 | 1.45 | -4.50 | 1.18 | -3.33 | 0.00 | 3.30 | -0.50 |
|  | SC vs TAU | 0.92 | 1.21 | | | -1.45 | | 3.29 | | -0.47 | | 2.31 | | 3.30 | | 0.28 | |  | | -1.44 | 1.38 | -4.14 | 1.27 | -3.03 | 0.15 | 3.30 | -0.44 |
|  | SC vs GiVE | 2.54 | 1.20 | | | 0.19 | | 4.90 | | 1.16 | | 3.93 | | 3.30 | | 0.77 | |  | | 0.22 | 1.43 | -2.59 | 3.03 | -1.43 | 1.87 | 3.30 | 0.07 |
| PSYRATS: Frequency | GiVE vs TAU | -0.51 | 0.59 | | | -1.67 | | 0.65 | | -1.19 | | 0.17 | | 2.00 | | -0.26 | |  | | 0.71 | 0.75 | -0.76 | 2.18 | -0.15 | 1.58 | 2.00 | 0.36 |
|  | SC vs TAU | 0.86 | 0.59 | | | -0.29 | | 2.01 | | 0.18 | | 1.54 | | 2.00 | | 0.43 | |  | | 0.35 | 0.70 | -1.02 | 1.73 | -0.45 | 1.16 | 2.00 | 0.18 |
|  | SC vs GiVE | 1.37 | 0.60 | | | 0.20 | | 2.54 | | 0.68 | | 2.05 | | 2.00 | | 0.69 | |  | | -0.36 | 0.74 | -1.81 | 1.09 | -1.21 | 0.49 | 2.00 | -0.18 |
| PSYRATS: Loudness | GiVE vs TAU | -0.01 | 0.26 | | | -0.52 | | 0.50 | | -0.31 | | 0.29 | | 0.97 | | -0.01 | |  | | -0.11 | 0.33 | -0.75 | 0.53 | -0.48 | 0.27 | 097 | -0.11 |
|  | SC vs TAU | 0.19 | 0.26 | | | -0.31 | | 0.69 | | -0.10 | | 0.48 | | 0.97 | | 0.20 | |  | | -0.44 | 0.30 | -1.04 | 0.15 | -0.79 | -0.09 | 0.97 | -0.46 |
|  | SC vs GiVE | 0.20 | 0.25 | | | -0.30 | | 0.69 | | -0.09 | | 0.49 | | 0.97 | | 0.21 | |  | | -0.33 | 0.32 | -0.95 | 0.29 | -0.70 | 0.03 | 0.97 | -0.34 |
| PSYRATS: Attribution | GiVE vs TAU | -1.04 | 0.56 | | | -2.14 | | 0.07 | | -1.69 | | -0.39 | | 1.62 | | -0.64 | |  | | -0.78 | 0.71 | -2.18 | 0.61 | -1.61 | 0.04 | 1.62 | -0.49 |
|  | SC vs TAU | -0.35 | 0.56 | | | -1.44 | | 0.74 | | -0.99 | | 0.29 | | 1.62 | | -0.22 | |  | | -0.06 | 0.66 | -1.35 | 1.22 | -0.82 | 0.69 | 1.62 | -0.04 |
|  | SC vs GiVE | 0.68 | 0.56 | | | -0.42 | | 1.78 | | 0.04 | | 1.33 | | 1.62 | | 0.42 | |  | | 0.72 | 0.69 | -0.63 | 2.07 | -0.07 | 1.51 | 1.62 | 0.45 |
| PSYRATS: Total | GiVE vs TAU | -2.86 | 2.11 | | | -6.99 | | 1.27 | | -5.29 | | -0.44 | | 5.58 | | -0.51 | |  | | -1.81 | 2.54 | -6.78 | 3.17 | -4.73 | 1.11 | 5.58 | -0.32 |
|  | SC vs TAU | 2.20 | 2.12 | | | -1.94 | | 6.35 | | -0.23 | | 4.64 | | 5.58 | | 0.39 | |  | | -0.78 | 2.49 | -5.65 | 4.10 | -3.64 | 2.08 | 5.58 | -0.14 |
|  | SC vs GiVE | 5.06 | 2.11 | | | 0.93 | | 9.20 | | 2.64 | | 7.49 | | 5.58 | | 0.91 | |  | | 1.03 | 2.56 | -3.99 | 6.06 | -1.92 | 3.98 | 5.58 | 0.19 |
| VIS: Negative Impact | GiVE vs TAU | 7.78 | 4.67 | | | -1.37 | | 16.93 | | 2.41 | | 13.15 | | 12.41 | | 0.63 | |  | | 6.12 | 5.34 | -4.33 | 16.58 | -0.02 | 12.26 | 12.41 | 0.49 |
|  | SC vs TAU | 2.50 | 4.84 | | | -6.98 | | 11.98 | | -3.07 | | 8.07 | | 12.41 | | 0.20 | |  | | 8.94 | 5.25 | -1.35 | 19.23 | 2.90 | 14.98 | 12.41 | 0.72 |
|  | SC vs GiVE | -5.28 | 4.86 | | | -14.81 | | 4.25 | | -10.87 | | 0.32 | | 12.41 | | -0.43 | |  | | 2.82 | 5.44 | -7.85 | 13.49 | -3.44 | 9.08 | 12.41 | 0.23 |
| VIS: Positive Impact | GiVE vs TAU | 4.06 | 3.67 | | | -3.14 | | 11.25 | | -0.17 | | 8.28 | | 16.96 | | 0.24 | |  | | 0.64 | 4.00 | -7.21 | 8.49 | -3.97 | 5.24 | 16.96 | 0.04 |
|  | SC vs TAU | -0.09 | 3.81 | | | -7.56 | | 7.38 | | -4.48 | | 4.29 | | 16.96 | | -0.01 | |  | | 5.13 | 4.06 | -2.83 | 13.10 | 0.46 | 9.81 | 16.96 | 0.30 |
|  | SC vs GiVE | -4.15 | 3.93 | | | -11.85 | | 3.55 | | -8.67 | | 0.37 | | 16.96 | | -0.24 | |  | | 4.49 | 4.19 | -3.71 | 12.70 | -0.32 | 9.31 | 16.96 | 0.26 |
| VIS: Living Well | GiVE vs TAU | 8.77 | 4.36 | | | 0.22 | | 17.33 | | 3.75 | | 13.79 | | 15.06 | | 0.58 | |  | | 1.92 | 5.23 | -8.32 | 12.17 | -4.09 | 7.94 | 15.06 | 0.13 |
|  | SC vs TAU | 3.27 | 4.29 | | | -5.15 | | 11.68 | | -1.67 | | 8.21 | | 15.06 | | 0.22 | |  | | 11.94 | 4.96 | 2.23 | 21.66 | 6.24 | 17.64 | 15.06 | 0.79 |
|  | SC vs GiVE | -5.51 | 4.49 | | | -14.31 | | 3.30 | | -10.68 | | -0.34 | | 15.06 | | -0.37 | |  | | 10.02 | 5.24 | -0.24 | 20.28 | 4.00 | 16.04 | 15.06 | 0.67 |
| VIS: Total | GiVE vs TAU | 21.04 | 8.90 | | | 3.59 | | 38.49 | | 10.80 | | 31.28 | | 34.52 | | 0.61 | |  | | 8.87 | 9.88 | -10.50 | 28.24 | -2.50 | 20.24 | 34.52 | 0.26 |
|  | SC vs TAU | 6.42 | 8.95 | | | -11.12 | | 23.97 | | -3.87 | | 16.72 | | 34.52 | | 0.19 | |  | | 25.05 | 9.75 | 5.95 | 44.15 | 13.84 | 36.26 | 34.52 | 0.73 |
|  | SC vs GiVE | -14.62 | 9.46 | | | -33.15 | | 3.92 | | -25.49 | | -3.74 | | 34.52 | | -0.42 | |  | | 16.18 | 10.23 | -3.88 | 36.23 | 4.41 | 27.95 | 34.52 | 0.47 |
| HADS: Depression | GiVE vs TAU | -2.11 | 1.03 | | | -4.12 | | -0.10 | | -3.29 | | -0.93 | | 3.80 | | -0.55 | |  | | -2.31 | 1.17 | -4.61 | -0.02 | -3.66 | -0.97 | 3.80 | -0.61 |
|  | SC vs TAU | -0.46 | 1.07 | | | -2.56 | | 1.64 | | -1.69 | | 0.77 | | 3.80 | | -0.12 | |  | | -1.44 | 1.17 | -3.73 | 0.85 | -2.78 | -0.09 | 3.80 | -0.38 |
|  | SC vs GiVE | 1.65 | 1.08 | | | -0.46 | | 3.76 | | 0.41 | | 2.89 | | 3.80 | | 0.43 | |  | | 0.88 | 1.22 | -1.51 | 3.26 | -0.52 | 2.27 | 3.80 | 0.23 |
| HADS: Anxiety | GiVE vs TAU | -0.92 | 1.06 | | | -2.98 | | 1.15 | | -2.13 | | 0.30 | | 4.03 | | -0.23 | |  | | -1.77 | 1.32 | -4.35 | 0.81 | -3.28 | -0.26 | 4.03 | -0.44 |
|  | SC vs TAU | -0.24 | 1.06 | | | -2.32 | | 1.85 | | -1.46 | | 0.99 | | 4.03 | | -0.06 | |  | | -0.86 | 1.25 | -3.31 | 1.59 | -2.30 | 0.58 | 4.03 | -0.21 |
|  | SC vs GiVE | 0.68 | 1.06 | | | -1.41 | | 2.76 | | -0.54 | | 1.90 | | 4.03 | | 0.17 | |  | | 0.91 | 1.32 | -1.68 | 3.50 | -0.61 | 2.43 | 4.03 | 0.23 |
| CHOICE-SF: Mean | GiVE vs TAU | 1.59 | 0.48 | | | 0.65 | | 2.53 | | 1.04 | | 2.14 | | 1.55 | | 1.03 | |  | | 0.95 | 0.58 | -0.18 | 2.08 | 0.29 | 1.61 | 1.55 | 0.62 |
|  | SC vs TAU | 1.12 | 0.47 | | | 0.19 | | 2.05 | | 0.58 | | 1.67 | | 1.55 | | 0.73 | |  | | 1.04 | 0.55 | -0.02 | 2.11 | 0.42 | 1.67 | 1.55 | 0.68 |
|  | SC vs GiVE | -0.46 | 0.48 | | | -1.40 | | 0.47 | | -1.01 | | 0.08 | | 1.55 | | -0.30 | |  | | 0.09 | 0.57 | -1.03 | 1.21 | -0.56 | 0.75 | 1.55 | 0.06 |
| CHOICE-SF: Goal Rating | GiVE vs TAU | 2.75 | 0.78 | | | 1.23 | | 4.28 | | 1.86 | | 3.65 | | 2.50 | | 1.10 | |  | | 2.30 | 0.97 | 0.40 | 4.19 | 1.19 | 3.41 | 2.50 | 0.92 |
|  | SC vs TAU | 1.42 | 0.77 | | | -0.08 | | 2.92 | | 0.54 | | 2.30 | | 2.50 | | 0.57 | |  | | 2.06 | 0.91 | 0.27 | 3.85 | 1.01 | 3.11 | 2.50 | 0.82 |
|  | SC vs GiVE | -1.34 | 0.76 | | | -2.83 | | 0.16 | | -2.21 | | -0.46 | | 2.50 | | -0.53 | |  | | -0.24 | 0.94 | -2.08 | 1.60 | -1.32 | 0.84 | 2.50 | -0.10 |
| BCSS: Negative Self | GiVE vs TAU | -3.34 | 1.45 | | | -6.19 | | -0.50 | | -5.01 | | -1.67 | | 6.08 | | -0.55 | |  | | -0.61 | 1.69 | -3.93 | 2.71 | -2.56 | 1.34 | 6.08 | -0.10 |
|  | SC vs TAU | -4.84 | 1.40 | | | -7.57 | | -2.10 | | -6.44 | | -3.23 | | 6.08 | | -0.79 | |  | | -0.18 | 1.60 | -3.32 | 2.95 | -2.02 | 1.66 | 6.08 | -0.03 |
|  | SC vs GiVE | -1.49 | 1.41 | | | -4.25 | | 1.26 | | -3.11 | | 0.12 | | 6.08 | | -0.25 | |  | | 0.43 | 1.67 | -2.85 | 3.71 | -1.50 | 2.35 | 6.08 | 0.07 |
| BCSS: Positive Self | GiVE vs TAU | 3.11 | 1.34 | | | 0.49 | | 5.73 | | 1.57 | | 4.65 | | 4.93 | | 0.63 | |  | | 2.95 | 1.59 | -0.17 | 6.07 | 1.12 | 4.78 | 4.93 | 0.60 |
|  | SC vs TAU | 0.71 | 1.34 | | | -1.92 | | 3.34 | | -0.83 | | 2.25 | | 4.93 | | 0.14 | |  | | 1.84 | 1.56 | -1.22 | 4.90 | 0.05 | 3.64 | 4.93 | 0.37 |
|  | SC vs GiVE | -2.40 | 1.30 | | | -4.94 | | 0.15 | | -3.89 | | -0.90 | | 4.93 | | -0.49 | |  | | -1.11 | 1.59 | -4.22 | 2.01 | -2.94 | 0.72 | 4.93 | -0.22 |
| VAY: Voice Dominance | GiVE vs TAU | -1.88 | 1.25 | | | -4.34 | | 0.57 | | -3.32 | | -0.44 | | 4.72 | | -0.40 | |  | | -2.69 | 1.49 | -5.62 | 0.24 | -4.41 | -0.97 | 4.72 | -0.57 |
|  | SC vs TAU | -1.15 | 1.24 | | | -3.57 | | 1.27 | | -2.57 | | 0.27 | | 4.72 | | -0.24 | |  | | -3.71 | 1.42 | -6.50 | -0.92 | -5.35 | -2.08 | 4.72 | -0.79 |
|  | SC vs GiVE | 0.73 | 1.21 | | | -1.63 | | 3.10 | | -0.66 | | 2.12 | | 4.72 | | 0.16 | |  | | -1.02 | 1.48 | -3.91 | 1.87 | -2.72 | 0.68 | 4.72 | -0.22 |
| VAY: Voice Intrusiveness | GiVE vs TAU | -0.38 | 1.14 | | | -2.61 | | 1.85 | | -1.69 | | 0.93 | | 3.90 | | -0.10 | |  | | -0.22 | 1.38 | -2.93 | 2.49 | -1.81 | 1.37 | 3.90 | -0.06 |
|  | SC vs TAU | 1.06 | 1.12 | | | -1.14 | | 3.27 | | -0.23 | | 2.36 | | 3.90 | | 0.27 | |  | | -0.07 | 1.30 | -2.62 | 2.47 | -1.57 | 1.42 | 3.90 | -0.02 |
|  | SC vs GiVE | 1.44 | 1.13 | | | -0.78 | | 3.66 | | 0.14 | | 2.75 | | 3.90 | | 0.37 | |  | | 0.15 | 1.38 | -2.56 | 2.85 | -1.44 | 1.74 | 3.90 | 0.04 |
| VAY: Hearer Dependence | GiVE vs TAU | -0.04 | 1.36 | | | -2.70 | | 2.62 | | -1.60 | | 1.52 | | 5.65 | | -0.01 | |  | | 1.88 | 1.64 | -1.32 | 5.09 | 0.00 | 3.76 | 5.65 | 0.33 |
|  | SC vs TAU | -1.33 | 1.42 | | | -4.12 | | 1.46 | | -2.96 | | 0.31 | | 5.65 | | -0.24 | |  | | 1.83 | 1.62 | -1.34 | 5.00 | -0.03 | 3.69 | 5.65 | 0.32 |
|  | SC vs GiVE | -1.29 | 1.41 | | | -4.05 | | 1.47 | | -2.91 | | 0.33 | | 5.65 | | -0.23 | |  | | -0.05 | 1.65 | -3.29 | 3.19 | -1.95 | 1.85 | 5.65 | -0.01 |
| VAY: Hearer Distance | GiVE vs TAU | -0.72 | 1.05 | | | -2.79 | | 1.34 | | -1.94 | | 0.49 | | 4.59 | | -0.16 | |  | | -0.17 | 1.30 | -2.72 | 2.37 | -1.67 | 1.32 | 4.59 | -0.04 |
|  | SC vs TAU | 1.84 | 1.10 | | | -0.31 | | 4.00 | | 0.58 | | 3.11 | | 4.59 | | 0.40 | |  | | -0.08 | 1.27 | -2.57 | 2.42 | -1.54 | 1.39 | 4.59 | -0.02 |
|  | SC vs GiVE | 2.57 | 1.07 | | | 0.46 | | 4.67 | | 1.33 | | 3.80 | | 4.59 | | 0.56 | |  | | 0.10 | 1.30 | -2.44 | 2.64 | -1.39 | 1.59 | 4.59 | 0.02 |
| BAVQ: Persecutory | GiVE vs TAU | -2.51 | 1.51 | | | -5.47 | | 0.46 | | -4.25 | | -0.77 | | 6.05 | | -0.41 | |  | | -1.92 | 1.86 | -5.56 | 1.72 | -4.05 | 0.22 | 6.05 | -0.32 |
|  | SC vs TAU | -0.37 | 1.50 | | | -3.31 | | 2.57 | | -2.10 | | 1.35 | | 6.05 | | -0.06 | |  | | -0.03 | 1.77 | -3.50 | 3.44 | -2.07 | 2.00 | 6.05 | -0.01 |
|  | SC vs GiVE | 2.13 | 1.50 | | | -0.82 | | 5.08 | | 0.40 | | 3.86 | | 6.05 | | 0.35 | |  | | 1.89 | 1.85 | -1.74 | 5.52 | -0.24 | 4.02 | 6.05 | 0.31 |
| BAVQ: Benevolence | GiVE vs TAU | 0.60 | 0.78 | | | -0.91 | | 2.12 | | -0.29 | | 1.50 | | 3.52 | | 0.17 | |  | | 1.13 | 0.84 | -0.52 | 2.78 | 0.16 | 2.10 | 3.52 | 0.32 |
|  | SC vs TAU | 0.72 | 0.79 | | | -0.83 | | 2.27 | | -0.19 | | 1.63 | | 3.52 | | 0.20 | |  | | 0.73 | 0.84 | -0.91 | 2.37 | -0.23 | 1.70 | 3.52 | 0.21 |
|  | SC vs GiVE | 0.11 | 0.79 | | | -1.44 | | 1.67 | | -0.80 | | 1.03 | | 3.52 | | 0.03 | |  | | -0.39 | 0.86 | -2.07 | 1.29 | -1.38 | 0.59 | 3.52 | -0.11 |
| PROQ3: UN Negative | GiVE vs TAU | -0.73 | 0.85 | | | -2.39 | | 0.93 | | -1.71 | | 0.24 | | 4.00 | | -0.18 | |  | | -0.38 | 1.09 | -2.52 | 1.77 | -1.64 | 0.88 | 4.00 | -0.09 |
|  | SC vs TAU | -0.30 | 0.84 | | | -1.95 | | 1.34 | | -1.27 | | 0.66 | | 4.00 | | -0.08 | |  | | -0.51 | 1.04 | -2.54 | 1.52 | -1.70 | 0.69 | 4.00 | -0.13 |
|  | SC vs GiVE | 0.43 | 0.85 | | | -1.23 | | 2.09 | | -0.55 | | 1.40 | | 4.00 | | 0.11 | |  | | -0.13 | 1.09 | -2.27 | 2.02 | -1.39 | 1.13 | 4.00 | -0.03 |
| PROQ3: UN Positive | GiVE vs TAU | 0.15 | 0.32 | | | -0.47 | | 0.78 | | -0.21 | | 0.52 | | 1.19 | | 0.13 | |  | | 0.09 | 0.39 | -0.68 | 0.87 | -0.36 | 0.55 | 1.19 | 0.08 |
|  | SC vs TAU | -0.18 | 0.32 | | | -0.81 | | 0.45 | | -0.55 | | 0.19 | | 1.19 | | -0.15 | |  | | -0.18 | 0.38 | -0.93 | 0.57 | -0.62 | 0.26 | 1.19 | -0.15 |
|  | SC vs GiVE | -0.33 | 0.33 | | | -0.98 | | 0.31 | | -0.71 | | 0.05 | | 1.19 | | -0.28 | |  | | -0.27 | 0.41 | -1.07 | 0.52 | -0.74 | 0.19 | 1.19 | -0.23 |
| PROQ3: UC Negative | GiVE vs TAU | 0.50 | 0.87 | | | -1.20 | | 2.20 | | -0.49 | | 1.50 | | 3.52 | | 0.14 | |  | | 1.92 | 1.10 | -0.23 | 4.07 | 0.65 | 3.18 | 3.52 | 0.54 |
|  | SC vs TAU | 0.58 | 0.86 | | | -1.10 | | 2.25 | | -0.41 | | 1.56 | | 3.52 | | 0.16 | |  | | 0.89 | 1.04 | -1.15 | 2.93 | -0.31 | 2.09 | 3.52 | 0.25 |
|  | SC vs GiVE | 0.07 | 0.87 | | | -1.62 | | 1.77 | | -0.92 | | 1.07 | | 3.52 | | 0.02 | |  | | -1.03 | 1.10 | -3.17 | 1.12 | -2.29 | 0.24 | 3.52 | -0.29 |
| PROQ3: UC Positive | GiVE vs TAU | -0.03 | 0.22 | | | -0.46 | | 0.41 | | -0.28 | | 0.23 | | 0.92 | | -0.03 | |  | | 0.10 | 0.29 | -0.46 | 0.67 | -0.23 | 0.44 | 0.92 | 0.11 |
|  | SC vs TAU | 0.16 | 0.22 | | | -0.26 | | 0.59 | | -0.09 | | 0.42 | | 0.92 | | 0.18 | |  | | 0.07 | 0.27 | -0.46 | 0.61 | -0.24 | 0.39 | 0.92 | 0.08 |
|  | SC vs GiVE | 0.19 | 0.22 | | | -0.24 | | 0.62 | | -0.06 | | 0.45 | | 0.92 | | 0.21 | |  | | -0.03 | 0.29 | -0.60 | 0.53 | -0.36 | 0.30 | 0.92 | -0.03 |
| PROQ3: NC Negative | GiVE vs TAU | 0.02 | 0.90 | | | -1.76 | | 1.79 | | -1.02 | | 1.06 | | 4.43 | | 0.00 | |  | | 0.27 | 1.14 | -1.96 | 2.51 | -1.04 | 1.58 | 4.43 | 0.06 |
|  | SC vs TAU | 1.59 | 0.89 | | | -0.16 | | 3.34 | | 0.56 | | 2.62 | | 4.43 | | 0.36 | |  | | -0.28 | 1.08 | -2.40 | 1.84 | -1.52 | 0.96 | 4.43 | -0.06 |
|  | SC vs GiVE | 1.58 | 0.90 | | | -0.19 | | 3.35 | | 0.54 | | 2.62 | | 4.43 | | 0.36 | |  | | -0.55 | 1.14 | -2.78 | 1.68 | -1.86 | 0.76 | 4.43 | -0.12 |
| PROQ3: NC Positive | GiVE vs TAU | 0.28 | 0.32 | | | -0.35 | | 0.91 | | -0.09 | | 0.65 | | 1.34 | | 0.21 | |  | | 0.11 | 0.39 | -0.65 | 0.87 | -0.34 | 0.56 | 1.34 | 0.08 |
|  | SC vs TAU | 0.25 | 0.32 | | | -0.38 | | 0.87 | | -0.12 | | 0.61 | | 1.34 | | 0.19 | |  | | 0.17 | 0.37 | -0.55 | 0.90 | -0.25 | 0.60 | 1.34 | 0.13 |
|  | SC vs GiVE | -0.03 | 0.32 | | | -0.66 | | 0.59 | | -0.40 | | 0.34 | | 1.34 | | -0.03 | |  | | 0.06 | 0.39 | -0.70 | 0.82 | -0.38 | 0.51 | 1.34 | 0.05 |
| PROQ3: LC Negative | GiVE vs TAU | -1.82 | 0.93 | | | -3.64 | | -0.01 | | -2.89 | | -0.76 | | 4.05 | | -0.45 | |  | | -0.72 | 1.18 | -3.04 | 1.60 | -2.08 | 0.64 | 4.05 | -0.18 |
|  | SC vs TAU | -0.97 | 0.91 | | | -2.75 | | 0.82 | | -2.02 | | 0.08 | | 4.05 | | -0.24 | |  | | -1.93 | 1.12 | -4.12 | 0.25 | -3.22 | -0.65 | 4.05 | -0.48 |
|  | SC vs GiVE | 0.86 | 0.91 | | | -0.94 | | 2.65 | | -0.20 | | 1.91 | | 4.05 | | 0.21 | |  | | -1.21 | 1.17 | -3.51 | 1.09 | -2.56 | 0.14 | 4.05 | -0.30 |
| PROQ3: LC Positive | GiVE vs TAU | -0.07 | 0.29 | | | -0.63 | | 0.50 | | -0.40 | | 0.26 | | 1.01 | | -0.07 | |  | | -0.47 | 0.36 | -1.18 | 0.24 | -0.89 | -0.06 | 1.01 | -0.46 |
|  | SC vs TAU | -0.21 | 0.29 | | | -0.77 | | 0.35 | | -0.54 | | 0.12 | | 1.01 | | -0.20 | |  | | -0.41 | 0.35 | -1.09 | 0.27 | -0.81 | -0.01 | 1.01 | -0.40 |
|  | SC vs GiVE | -0.14 | 0.29 | | | -0.71 | | 0.43 | | -0.47 | | 0.19 | | 1.01 | | -0.14 | |  | | 0.06 | 0.36 | -0.65 | 0.78 | -0.36 | 0.48 | 1.01 | 0.06 |
| PROQ3: LN Negative | GiVE vs TAU | -0.95 | 0.96 | | | -2.83 | | 0.94 | | -2.05 | | 0.16 | | 4.40 | | -0.21 | |  | | 0.06 | 1.20 | -2.29 | 2.40 | -1.32 | 1.43 | 4.40 | 0.01 |
|  | SC vs TAU | -0.12 | 0.95 | | | -1.98 | | 1.74 | | -1.22 | | 0.97 | | 4.40 | | -0.03 | |  | | 0.67 | 1.13 | -1.55 | 2.90 | -0.63 | 1.98 | 4.40 | 0.15 |
|  | SC vs GiVE | 0.82 | 0.96 | | | -1.06 | | 2.70 | | -0.28 | | 1.93 | | 4.40 | | 0.19 | |  | | 0.62 | 1.19 | -1.72 | 2.95 | -0.76 | 1.99 | 4.40 | 0.14 |
| PROQ3: LN Positive | GiVE vs TAU | 0.09 | 0.29 | | | -0.48 | | 0.65 | | -0.25 | | 0.42 | | 1.21 | | 0.07 | |  | | -0.04 | 0.35 | -0.73 | 0.64 | -0.45 | 0.36 | 1.21 | -0.04 |
|  | SC vs TAU | 0.10 | 0.29 | | | -0.47 | | 0.66 | | -0.23 | | 0.43 | | 1.21 | | 0.08 | |  | | 0.39 | 0.34 | -0.26 | 1.05 | 0.01 | 0.78 | 1.21 | 0.33 |
|  | SC vs GiVE | 0.01 | 0.29 | | | -0.55 | | 0.57 | | -0.32 | | 0.34 | | 1.21 | | 0.01 | |  | | 0.44 | 0.35 | -0.26 | 1.13 | 0.03 | 0.85 | 1.21 | 0.36 |
| PROQ3: LD Negative | GiVE vs TAU | -0.49 | 0.85 | | | -2.16 | | 1.17 | | -1.47 | | 0.48 | | 3.98 | | -0.12 | |  | | -1.16 | 1.04 | -3.20 | 0.88 | -2.36 | 0.04 | 3.98 | -0.29 |
|  | SC vs TAU | -0.42 | 0.84 | | | -2.07 | | 1.23 | | -1.39 | | 0.55 | | 3.98 | | -0.11 | |  | | -0.61 | 0.99 | -2.55 | 1.34 | -1.75 | 0.53 | 3.98 | -0.15 |
|  | SC vs GiVE | 0.08 | 0.85 | | | -1.59 | | 1.74 | | -0.90 | | 1.05 | | 3.98 | | 0.02 | |  | | 0.55 | 1.04 | -1.49 | 2.59 | -0.65 | 1.75 | 3.98 | 0.14 |
| PROQ3: LD Positive | GiVE vs TAU | -0.04 | 0.31 | | | -0.65 | | 0.58 | | -0.40 | | 0.32 | | 1.20 | | -0.03 | |  | | 0.35 | 0.38 | -0.39 | 1.10 | -0.08 | 0.79 | 1.20 | 0.29 |
|  | SC vs TAU | -0.63 | 0.31 | | | -1.23 | | -0.02 | | -0.98 | | -0.27 | | 1.20 | | -0.52 | |  | | 0.13 | 0.36 | -0.57 | 0.84 | -0.28 | 0.55 | 1.20 | 0.11 |
|  | SC vs GiVE | -0.59 | 0.31 | | | -1.20 | | 0.03 | | -0.95 | | -0.23 | | 1.20 | | -0.49 | |  | | -0.22 | 0.38 | -0.96 | 0.52 | -0.66 | 0.21 | 1.20 | -0.18 |
| PROQ3: ND Negative | GiVE vs TAU | 0.07 | 0.83 | | | -1.57 | | 1.70 | | -0.89 | | 1.03 | | 4.10 | | 0.02 | |  | | -0.62 | 1.03 | -2.63 | 1.40 | -1.80 | 0.57 | 4.10 | -0.15 |
|  | SC vs TAU | -1.51 | 0.82 | | | -3.12 | | 0.10 | | -2.46 | | -0.57 | | 4.10 | | -0.37 | |  | | -0.81 | 0.98 | -2.73 | 1.11 | -1.94 | 0.31 | 4.10 | -0.20 |
|  | SC vs GiVE | -1.58 | 0.83 | | | -3.20 | | 0.04 | | -2.53 | | -0.63 | | 4.10 | | -0.39 | |  | | -0.19 | 1.03 | -2.21 | 1.82 | -1.37 | 0.99 | 4.10 | -0.05 |
| PROQ3: ND Positive | GiVE vs TAU | -0.18 | 0.31 | | | -0.78 | | 0.41 | | -0.54 | | 0.17 | | 1.13 | | -0.16 | |  | | -0.15 | 0.38 | -0.90 | 0.59 | -0.59 | 0.28 | 1.13 | -0.13 |
|  | SC vs TAU | -0.37 | 0.32 | | | -0.99 | | 0.24 | | -0.74 | | -0.01 | | 1.13 | | -0.33 | |  | | -0.39 | 0.37 | -1.11 | 0.33 | -0.81 | 0.03 | 1.13 | -0.35 |
|  | SC vs GiVE | -0.19 | 0.32 | | | -0.81 | | 0.43 | | -0.55 | | 0.17 | | 1.13 | | -0.17 | |  | | -0.24 | 0.39 | -1.00 | 0.52 | -0.69 | 0.21 | 1.13 | -0.21 |
| PROQ3: UD Negative | GiVE vs TAU | 0.60 | 0.71 | | | -0.80 | | 2.00 | | -0.22 | | 1.42 | | 3.99 | | 0.15 | |  | | 2.32 | 0.89 | 0.58 | 4.05 | 1.30 | 3.33 | 3.99 | 0.58 |
|  | SC vs TAU | 1.18 | 0.70 | | | -0.19 | | 2.55 | | 0.38 | | 1.99 | | 3.99 | | 0.30 | |  | | 0.94 | 0.84 | -0.70 | 2.58 | -0.03 | 1.90 | 3.99 | 0.23 |
|  | SC vs GiVE | 0.58 | 0.71 | | | -0.80 | | 1.97 | | -0.23 | | 1.40 | | 3.99 | | 0.15 | |  | | -1.38 | 0.88 | -3.11 | 0.35 | -2.39 | -0.36 | 3.99 | -0.35 |
| PROQ3: UD Positive | GiVE vs TAU | 0.38 | 0.32 | | | -0.24 | | 1.00 | | 0.02 | | 0.75 | | 1.32 | | 0.29 | |  | | 0.14 | 0.39 | -0.62 | 0.91 | -0.31 | 0.60 | 1.32 | 0.11 |
|  | SC vs TAU | 0.51 | 0.31 | | | -0.10 | | 1.13 | | 0.15 | | 0.87 | | 1.32 | | 0.39 | |  | | 0.57 | 0.38 | -0.16 | 1.31 | 0.14 | 1.00 | 1.32 | 0.43 |
|  | SC vs GiVE | 0.13 | 0.31 | | | -0.48 | | 0.74 | | -0.22 | | 0.49 | | 1.32 | | 0.10 | |  | | 0.43 | 0.39 | -0.34 | 1.19 | -0.02 | 0.88 | 1.32 | 0.32 |
| PROQ3: Total | GiVE vs TAU | -2.03 | 4.14 | | | -10.14 | | 6.08 | | -6.79 | | 2.73 | | 19.25 | | -0.11 | |  | | 2.05 | 5.20 | -8.14 | 12.24 | -3.93 | 8.03 | 19.25 | 0.11 |
|  | SC vs TAU | 0.01 | 4.10 | | | -8.03 | | 8.04 | | -4.71 | | 4.72 | | 19.25 | | 0.00 | |  | | -0.78 | 4.92 | -10.43 | 8.87 | -6.45 | 4.88 | 19.25 | -0.04 |
|  | SC vs GiVE | 2.04 | 4.09 | | | -5.99 | | 10.06 | | -2.67 | | 6.75 | | 19.25 | | 0.11 | |  | | -2.84 | 5.18 | -13.00 | 7.32 | -8.80 | 3.13 | 19.25 | -0.15 |
| PTS: Social Reference | GiVE vs TAU | -1.25 | 3.94 | | | -8.98 | | 6.47 | | -5.78 | | 3.28 | | 16.87 | | -0.07 | |  | | 2.33 | 4.62 | -6.72 | 11.38 | -2.98 | 7.64 | 16.87 | 0.14 |
|  | SC vs TAU | -0.75 | 3.91 | | | -8.41 | | 6.92 | | -5.24 | | 3.75 | | 16.87 | | -0.04 | |  | | 1.80 | 4.43 | -6.88 | 10.48 | -3.30 | 6.89 | 16.87 | 0.11 |
|  | SC vs GiVE | 0.51 | 3.89 | | | -7.11 | | 8.13 | | -3.96 | | 4.98 | | 16.87 | | 0.03 | |  | | -0.53 | 4.60 | -9.54 | 8.48 | -5.82 | 4.76 | 16.87 | -0.03 |
| PTS: Occupational Functioning | GiVE vs TAU | 1.48 | 4.67 | | | -7.67 | | 10.63 | | -3.89 | | 6.85 | | 18.52 | | 0.08 | |  | | 6.54 | 5.58 | -4.40 | 17.48 | 0.12 | 12.96 | 18.52 | 0.35 |
|  | SC vs TAU | 3.05 | 4.63 | | | -6.02 | | 12.12 | | -2.28 | | 8.37 | | 18.52 | | 0.16 | |  | | 1.03 | 5.34 | -9.43 | 11.49 | -5.11 | 7.17 | 18.52 | 0.06 |
|  | SC vs GiVE | 1.57 | 4.61 | | | -7.47 | | 10.61 | | -3.74 | | 6.88 | | 18.52 | | 0.08 | |  | | -5.51 | 5.56 | -16.41 | 5.38 | -11.91 | 0.88 | 18.52 | -0.30 |
| WSAS: Total | GiVE vs TAU | -0.67 | 2.18 | | | -4.94 | | 3.60 | | -3.18 | | 1.84 | | 7.79 | | -0.09 | |  | | -6.62 | 2.51 | -11.53 | -1.71 | -9.50 | -3.74 | 7.79 | -0.85 |
|  | SC vs TAU | -0.65 | 2.15 | | | -4.87 | | 3.56 | | -3.13 | | 1.82 | | 7.79 | | -0.08 | |  | | -1.58 | 2.36 | -6.20 | 3.04 | -4.29 | 1.13 | 7.79 | -0.20 |
|  | SC vs GiVE | 0.02 | 2.15 | | | -4.19 | | 4.23 | | -2.45 | | 2.49 | | 7.79 | | 0.00 | |  | | 5.04 | 2.46 | 0.21 | 9.87 | 2.21 | 7.87 | 7.79 | 0.65 |
| SOFAS: Total | GiVE vs TAU | 5.88 | 3.26 | | | -0.51 | | 12.27 | | 2.13 | | 9.63 | | 11.86 | | 0.50 | |  | | 6.22 | 3.83 | -1.29 | 13.73 | 1.81 | 10.62 | 11.86 | 0.52 |
|  | SC vs TAU | 7.22 | 3.19 | | | 0.96 | | 13.47 | | 3.54 | | 10.89 | | 11.86 | | 0.61 | |  | | 10.85 | 3.56 | 3.88 | 17.82 | 6.76 | 14.94 | 11.86 | 0.91 |
|  | SC vs GiVE | 1.34 | 3.21 | | | -4.96 | | 7.63 | | -2.36 | | 5.03 | | 11.86 | | 0.11 | |  | | 4.63 | 3.77 | -2.76 | 12.02 | 0.29 | 8.97 | 11.86 | 0.39 |

Notes: SE=standard error; LCL=lower confidence limit; UCL=upper confidence limit; SD=standard deviation; HPSVQ**=**Hamilton Program for Schizophrenia Voices Questionnaire; PSYRATS=Psychotic Symptoms Rating Scales; VIS=Voice Impact Scale; HADS=Hospital Anxiety and Depression Scale; CHOICE-SF=CHoice of Outcome In Cbt for psychosEs-Short Form ; BCSS=Brief Core Schema Scale; VAY = Voices And You; BAVQ=Beliefs About Voices Questionnaire; PROQ=Persons Relating to Others Questionnaire (UN=Upper Neutral, UC=Upper Close, NC, Neutral Close, LC, Lower Close, LN, Lower Neutral, LD, Lower Distant, ND=Neutral Distant, UD=Upper Distant) PTS=Paranoid Thoughts Scale; WSAS=Work and Social Adjustment Scale**;** SOFAS**=**Social and Occupational Functioning Scale; GiVE=Guided self-help CBT intervention for voices; TAU= Treatment as Usual; SC=Supportive Counselling; pairwise comparison group sizes can be taken from the Supplementary Table displaying the Descriptive Summary of Secondary Outcomes by Group and Time point
