# Supplementary material for: Increasing access to cognitive–behavioural therapy for patients with psychosis by evaluating the feasibility of a randomised controlled trial of brief, targeted cognitive–behavioural therapy for distressing voices delivered by assistant psychologists: the GiVE2 trial
Source: BJPsych Open. 2021 Aug 19;7(5):e152. doi: 10.1192/bjo.2021.983 (PMC8388007; doi:10.1192/bjo.2021.983)
Supplement: Supplementary file 1 [file bjosup.zip › S2056472421009832sup002.docx]

**Supplementary Information - Health Economic Analysis**

Contents

Aims and methods Page 2

Results Page 2

Conclusions Page 4

Appendix Page 11

Tables

Table 1: Sample sizes Page 2

Table 2: Service utilisation Page 6

Table 3: Costs of service use reported by participants Page 9

Table 4: Changes in numbers of medications Page 10

Table 5: Quality of Life, EQ-5D-5L Page 10

Aims and Methods

The aim of the health economic analysis was to assess the feasibility of gathering data for a cost-effectiveness analysis in a subsequent definitive trial. The health economic analysis set out to:

1. Assess the resource requirements and costs of the delivering the GiVE intervention and the Supportive Counselling (SC) intervention
2. Collect service use data from participants in all three arms (through interview conducted by researchers) using the Client Service Receipt Inventory (CSRI) [1] at 16 weeks (T1) and 28 weeks (T2), assess completeness and cross check data with mental health records to assess discrepancies
3. Convert service use to costs and compare between arms.
4. Assess health related quality of life using EQ-5D-5L [2] and SF-12 [3] at T0, T1 and T2, including data completeness and calculating Quality Adjusted Life Years (QALY) differences between the three arms. SF-12 was included in addition to EQ-5D-5L to see if SF-12 was more sensitive to psychological outcomes than EQ-5D-5L.

Resources and service use were converted to costs using nationally validated tariffs for staff time and consultations (see Appendix 1).

Results

Data available for the health economic analysis are shown in Table 1

***Table 1: Sample sizes***

| **Time period** | **Site** | | **All** | **TAU** | **GIVE** | **SC** |
| --- | --- | --- | --- | --- | --- | --- |
|  | **Pennine** | **Sussex** |  |  |  |  |
| **T0** | 45 | 34 | **79** | 27 | 26 | 26 |
| **T1** | 38 | 30 | **68** | 23 | 22 | 23 |
| **T2** | 25 | 20 | **45** | 16 | 12 | 17 |
| **Total** | 108 | 84 | **192** | 66 | 60 | 66 |

*Intervention resource requirements*

The GIVE intervention comprised 8 x 1 hour sessions per participant, delivered by Band 5 Clinical Psychologist assistant practitioner / entry level counsellor @£34 / hour (i.e. £272); materials (at retail price, data from study logs) comprised a workbook @ £5, and a self-help book @ £13, giving an overall total of £290/ participant

The SC intervention comprised 8 x 1 hour sessions per participant, delivered by Band 5 Clinical Psychologist assistant practitioner / entry level counsellor @£34 / hour i.e.£272 / participant

The threshold for therapy exposure was set at 6 of the 8 sessions – this was achieved by 23 GIVE and 19 SC participants. Individual level data on attendances were not available.

*CSRI: weeks 1 - 16 and 17 - 28*

Very little service use was reported for many items (see Table 2 below). The most frequently used were: outpatient psychiatric appointments, other outpatient including A&E, primary care (GP) contacts, district nurse contacts, community nurse/ case manager contacts. Comparison of usage across study arms for each of these five items at for the T0 to T1, and the T1 to T2 periods using Kruskal Wallis test found no significant differences, except for the use of community psychiatric nurse / case manager in the follow up time period when participants in the SC arm reported significantly higher contacts than the participants in the other two arms (p=0.017). A total of 21 contacts were reported by 7 of the 15 participants in TAU; 8 had zero contacts. Six of the 12 participants in the GIVE arm reported a total of 69 contacts, one outlier reporting 45 and 6 having zero contacts. Participants in the SC arm reported 80 contacts across 13 people, only 2 having zero contacts. A small number of participants in each group reported high amounts of home help / personal care input.

*Service Use Costs*

Costs of service use were calculated at the participant level (see Table 3 below). Costs of individual service use items were computed as the product of number of contacts and the unit cost using validated national tariffs. For community-based day services and primary and community care contacts, participants had reported the average duration of attendance and contact time and these were used in the calculation of the costs, rather than the standard national durations. Costs for items at individual level were summed for T0 to T1 and T1 to T2 separately, with intervention costs included for GiVE (£290 per participant) and SC (£272 per participant). Group means were estimated for each time period by item of service use and overall, and group means were compared. Participants with data for both time periods (T) to T1 and T1 to T2) were included in a comparison of costs by group for the entire study period.

Costs distribution was heavily skewed so analysis focused on group medians. During the intervention period (T0 to T1), excluding the cost of the interventions, the cost of the service use reported by the GIVE group was approximately double that of the SC and TAU groups. Adding in the intervention costs, there was a significant difference between the total costs of the groups (Kruskal Wallis p=0.006) reflecting group medians TAU £306.00, GiVE £897.50, SC £548.00). Amongst participants retained to T2, the GIVE group remained the most costly but the gap between groups had narrowed and was no longer significant. Table 3 for costs – see below

*CSRI data completeness*

T0 to T1: 65 of 68 participants provided data. Amongst those 65, completeness of the 22 items of the CSRI was very high. Of a total of 1430 possible items, n=16 (0.01%) were missing.

Weeks 17-28: 42 of 45 participants provided data. Amongst those 42, completeness of the 22 items of the CSRI was very high. Of a total of 924 possible items, n=11 (0.01%) were missing.

*Medications - Completeness of medication data*

|  | **T0** | **T1** | **T2** |
| --- | --- | --- | --- |
| **Pennine** | 44/45 | 20/38 | 6/26 |
| **Sussex** | 34/34 | 30/30 | 17/20 |

There were no changes in the number of medications reported by most patients during the study period (Table 4 – see below).

*Cross checking CSRI and records from mental health services*

This was done for medications data only as there was no way to check other service use for physical health in mental health service notes. The CSRI data on medications were checked for 6 randomly selected cases (by statistician) with the mental health service notes in each site. The agreement was 50% in Pennine and 59% in Sussex.

*Quality of life*

*EQ-5D-5L*

EQ-5D-5L data were available to enable assessment of change over the period of the intervention (T0 – T1) for 67 participants; changes over the follow up period (T1 – T2) could be assessed for 45 participants.

There was an improving trend in EQ-5D-5L group mean values from baseline to 16 weeks for all three groups. For participants with a follow up to 28 weeks, the improvement continued in the TAU and GIVE groups, but the mean value of the SC group fell to below its baseline level.

Converting EQ-5D-5L scores to utility values, the improvements in QALYs in the three groups between T0 and T1, and between T0 and T2 were (respectively): TAU 0.134, 0.258; GIVE 0.183, 0.350; SC 0.178, 0.309.

Regression modelling the increments in QALYs for the GIVE and SC groups, compared to TAU, and controlling for baseline utility values, revealed no significant differences between the groups (Table 5 – see below).

*SF-12*

One item (SF2 for the Physical Functioning domain) was missing from the SF-12 questionnaire at the beginning of the study due to a transcription error. As soon as this was noticed, it was corrected. Coupled with the lack of data for some participants at T2, this omission meant there was little SF-12 data available for analysis and the planned calculation of the SF-6 utility values from SF-12 health state responses [4,5] could not be completed for most participants at either time period. Missing SF-12 data was 53% at T0, 92% at T1 and 100% at T2. Moreover, with so few available observations, imputation was not possible. The mean SF-12 scores for the participants for which data were available at T0 and T1 were (respectively): TAU 0.513, 0.403; GIVE 0.551, 0.461; SC 0.576, 0.810 (Range 0 – 100, best; population mean 50, SD 10).

Conclusions

During the intervention period, weeks 0-16, the average costs for the GIVE group were higher than TAU. There was no evidence that the cost of the intervention was offset through other service use savings. There were no differences between groups in changes in quality of life during the intervention period. Statistical comparisons were exploratory as the trial was not powered to look at comparisons between groups. The numbers in each group were too small for reliable comparisons between groups during the follow up period. The costs are reliant on self-report as cross checking with medical records was not possible, except for medication data where limited consistency was found.

Data collection was feasible and levels of missingness were relatively low indicating that a similar methodology could be used in a subsequent full trial of clinical and cost effectiveness. Since correspondence between EQ-5D-5L and SF-12 for calculating QALYs could not be tested in this study, it would be prudent to include both instruments in future studies.

**References**

1. Thornicroft G, Becker T, Knapp M, Knudsen HC, Schene A, Tansella, M. Client Service Receipt Inventory. European version. *International outcome measures in mental health (Chapter 9)*. 2006. Royal College of Psychiatrists.

Euroqol group. The EQ-5D <https://euroqol.org/eq-5d-instruments/> Accessed 1^st^ Feb 2021

Ware JE, Kosinki M, Keller SD. SF-12: *How to Score the SF-12 Physical and Mental Health Summary Scales*. 1995. The Health Institute, New England Medical Centre.

1. Brazier, JE, Roberts, JR. The estimation of a preference-based index from the SF-12. *Medical Care*, 2004; **42(9):**851-859
2. Brazier, JE, Rowen, D, Hanmer, J. Revised SF-6D scoring programmes: a summary of improvements. *PRO newsletter*, 2008; **40**:14-15

***Table 2: Service utilisation***

*Service use by group T1 (Baseline to 16 weeks)*

*Note: Frequencies of contacts are shown in italics. For example:*

- *5.2 Psychiatric outpatient appointments, baseline to 16 weeks, group A (TAU). Of 11 participants reporting using this service, 8 reported 1 appointment and 3 reported 3 appointments*
- *5.4 Psychiatrist contacts, weeks 16 to 28, group A (TAU). Of 4 participants reporting a psychiatrist contact, 2 reported 1 contact, 1 reported 2 contacts and 1 reported 3 contacts.*

| **Service** | **Group** | **A: TAU** | | | **B: GiVE** | | | **C: SC** | | | **All** | | |
| --- | --- | --- | --- | --- | --- | --- | --- | --- | --- | --- | --- | --- | --- |
| **5.1 INPATIENT** | **Units** | **N** | **n** | **%** | **N** | **n** | **%** | **N** | **n** | **%** | **N** | **n** | **%** |
| Acute inpatient ward | Admissions | 23 | 0 |  | 21 | 0 |  | 21 | 1 | 4.5 | 65 | 1 | 1.5 |
| Psychiatric rehabilitation ward | Admissions | 23 | 0 |  | 21 | 0 |  | 22 | 0 |  | 66 | 0 |  |
| Long stay ward | Admissions | 23 | 0 |  | 21 | 0 |  | 22 | 0 |  | 66 | 0 |  |
| Emergency crisis centre | Admissions (days) | 23 | 0 |  | 20 | 1 (1) | 4.8 | 22 | 0 |  | 65 | 0 | 1.5 |
| General medical ward | Admissions (days) | 22 | 1 (3) | 4.3 | 21 | 0 |  | 22 | 0 |  | 65 | 1 | 1.5 |
| **5.2 OUTPATIENT** |  |  |  |  |  |  |  |  |  |  |  |  |  |
| Psychiatric outpatient | Appointment | 23 | 11  *8x1; 3x3* | 47.8 | 21 | 8  *7x1; 1x3* | 38.1 | 22 | 6  *5x1; 1x3* | 27.2 | 66 | 25 | 37.9 |
| Other outpatient including A&E | Appointment | 20 | 6  *3x1;1x2,3,5* | 30.0 | 17 | 4  *2x1; 1x2,4* | 23.5 | 18 | 1  1x1 | 5.6 | 55 | 11 | 20.0 |
| Day hospital | Day | 23 | 1  *1x1* | 4.3 | 21 | 2  *1x1; 1x3* | 9.6 | 22 | 0 |  | 66 | 3 | 4.5 |
| **5.3 Community based day services** | | | | | | | | | | | | | |
| Community mental health service | Attendances | 23 | 1  *1x16* | 4.3 | 21 | 1  *1x3* | 4.8 | 22 | 2  *1x1; 1x30* | 9.0 | 66 | 4 | 6.0 |
| Day care centre | Attendances | 23 | 0 |  | 21 | 0 |  | 22 | 0 |  | 66 | 0 |  |
| Group therapy | Attendances | 23 | 1  *1x8* | 4.3 | 21 | 0 |  | 22 | 3  *2x12; 1x15* | 13.6 | 66 | 4 | 6.0 |
| Sheltered workshop | Attendances | 23 | 1  *1x3* | 4.3 | 21 | 0 |  | 22 | 0 |  | 66 | 1 | 1.5 |
| Specialist education | Attendances | 23 |  |  | 21 | 0 |  | 21 | 1  *1x1* | 4.5 | 66 | 1 | 11.5 |
| **5.4 Other primary and community contacts** | | | | | | | | | | | | | |
| Psychiatrist | Contacts | 23 | 9  *8x1; 1x2* | 39.1 | 21 | 4  *3x1; 1x6* | 19.1 | 22 | 6  *3x1; 3x2* | 27.2 | 66 | 19 | 28.8 |
| Psychologist | Contacts | 23 | 1  *1x6* | 4.3 | 21 | 3  *2x1; 1x4* | 14.3 | 22 |  |  | 66 | 4 | 6.0 |
| Primary care physician | Contacts | 23 | 11  *6x1; 3x2; 2x3* | 47.8 | 21 | 9  *1x1,4,6; 4x2; 2x5* | 42.9 | 22 | 9  *6x1; 1x4,5,6* | 40.8 | 66 | 29 | 43.8 |
| District nurse | Contacts | 23 | 8  *3x1; 1x2,4,10; 2x3* | 34.6 | 21 | 9  *3x1;*  *1x2,4,6,12; 2x3* | 43.0 | 22 | 8  *6x1; 1x2,4* | 36.3 | 66 | 25 | 37.8 |
| Community psychiatric nurse/ case manager | Contacts | 23 | 14  *2x1,6; 4x2;*  *1x3,4,8,9,10,12* | 60.9 | 21 | 15  *3x1,6; 2x2,3,4;*  *1x6,8,13* | 71.4 | 22 | 13  *1x1,2,5,10,12,15;*  *2x6; 5x2* | 59.1 | 66 | 42 | 63.6 |
| Social worker | Contacts | 23 | 0 |  | 21 | 1  *1x4* | 4.8 | 22 | 0 |  | 66 | 1 | 1.5 |
| Occupational therapist | Contacts | 23 | 3  *1x2,3,4* | 13.0 | 21 | 0 |  | 20 | 2  1x3,12 | 9.1 | 66 | 5 | 7.6 |
| Home help/ care worker | Contacts | 23 | 4  *1x3,8,90,315* | 17.4 | 21 | 6  *1x2,12,15,*  *90,150,192* | 28.6 | 22 | 2  *1x48,90* | 9.1 | 66 | 12 | 18.2 |
| **5.5 Criminal justice service** | | | | | | | | | | | | | |
| Contact - | No | 23 | 0 |  | 21 | 0 |  | 22 | 0 |  | 66 | 0 |  |

*Service use by group T2 (16 to 28 weeks)*

| **Service** | **Group** | **A: TAU** | | | **B: GiVE** | | | **C: SC** | | | **All** | | |
| --- | --- | --- | --- | --- | --- | --- | --- | --- | --- | --- | --- | --- | --- |
| **5.1 INPATIENT** | **Units** | **N** | **n** | **%** | **N** | **n** | **%** | **N** | **n** | **%** | **N** | **n** | **%** |
| Acute inpatient ward | Admissions | 15 | 0 |  | 12 | 0 |  | 15 | 0 |  | 43 | 0 |  |
| Psychiatric rehabilitation ward | Admissions | 15 | 0 |  | 12 | 0 |  | 15 | 0 |  | 43 | 0 |  |
| Long stay ward | Admissions | 15 | 0 |  | 12 | 0 |  | 15 | 0 |  | 43 | 0 |  |
| Emergency crisis centre | Admissions (days) | 15 | 0 |  | 11 | 1 (3) | 8.3 | 15 | 0 |  | 42 | 1 | 2.4 |
| General medical ward | Admissions (days) | 14 | 1 (3) | 6.3 | 12 |  |  | 15 | 0 |  | 42 | 1 | 2.4 |
| **5.2 OUTPATIENT** |  |  |  |  |  |  |  |  |  |  |  |  |  |
| Psychiatric outpatient | Appointment | 14 | 4  *2x1; 2x2* | 28.6 | 12 | 4  *3x1; 1x3* | 33.3 | 15 | 5  *5x1* | 33.3 | 42 | 13 | 31.7 |
| Other outpatient including A&E | Appointment | 13 | 3  *2x2; 1x3* | 23.1 | 9 | 3  *3x1* | 33.3 | 14 | 3  *1x3* | 21.4 | 36 | 9 | 25.0 |
| Day hospital | Day | 14 | 0 |  | 12 | 0 |  | 15 | 0 |  | 41 | 0 |  |
| **5.3 Community based day services** | | | | | | | | | | | | | |
| Community mental health service | Attendances | 15 | 2  *1x5; 1x16* | 13.4 | 12 | 2  *1x1; 1x9* | 16.6 | 15 | 1  *1x12* | 6.7 | 42 | 5 | 11.6 |
| Day care centre | Attendances | 15 | 1  *1x65* | 6.7 | 12 |  |  | 15 | 0 |  | 42 | 1 | 2.4 |
| Group therapy | Attendances | 15 | 1  *1x2* | 6.7 | 12 | 1  *1x7* | 8.3 | 15 | 1  *1x12* | 6.7 | 42 | 3 | 7.1 |
| Sheltered workshop | Attendances | 16 | 0 |  | 12 | 0 |  | 15 | 1  *1x24* | 6.7 | 42 | 1 | 2.4 |
| Specialist education | Attendances | 16 | 2  *1x1; 1x7* | 13.4 | 12 | 1  *1x9* | 8.3 | 15 | 1  *1x6* | 6.7 | 42 | 4 | 9.5 |
| **5.4 Other primary and community contacts** | | | | | | | | | | | | | |
| Psychiatrist | Contacts | 15 | 4  *2x1; 1x2,3* | 26.7 | 12 | 5  5x1 | 41.7 | 14 | 3  *3x1* | 21.4 | 41 | 12 | 29.3 |
| Psychologist | Contacts | 15 | 2  *1x11; 1x12* | 13.4 | 12 | 2  *1x1; 1x2* | 16.6 | 15 | 1  *1x2* | 6.7 | 42 | 5 | 11.6 |
| Primary care physician | Contacts | 15 | 3  *3x1* | 20.0 | 12 | 5  *3x1; 1x2,3* | 41.6 | 15 | 5  *4x1; 1x3* | 33.4 | 42 | 13 | 31.0 |
| District nurse | Contacts | 15 | 7  *1x1; 4x3; 2x6* | 46.7 | 12 | 3  *2x3; 1x6* | 25.0 | 15 | 3  *2x3; 1x6* | 20.0 | 42 | 13 | 31.0 |
| Community psychiatric nurse/ case manager | Contacts | 15 | 7  *1x1,4,6; 2x2,3* | 46.7 | 12 | 6  *3x1; 1x9,12,45* | 50.0 | 15 | 13  *3x2; 2x5; 4x6;*  *1x3,4,15,18* | 86.7 | 42 | 26 | 61.9 |
| Social worker | Contacts | 15 | 0 |  | 12 | 0 |  | 15 | 0 |  | 42 | 0 |  |
| Occupational therapist | Contacts | 15 | 0 |  | 12 | 0 |  | 15 | 1  *1x12* | 6.7 | 42 | 1 | 2.4 |
| Home help/ care worker | Contacts | 15 | 2  *1x1,2* | 13.4 | 12 | 0 |  | 15 | 1  *1x90* | 6.7 | 42 | 3 | 7.1 |
| **5.5 Criminal justice service** | | | | | | | | | | | | | |
| Contact - No |  | 15 | 0 |  | 12 | 0 |  | 15 | 0 |  | 42 | 0 |  |

***Table 3: Costs of service use reported by participants***

| **T1 (Baseline to 16 weeks)** | **A: TAU** | | | | **B: GiVE** | | | | **C: SC** | | | | **Kruskal Wallis test p** |
| --- | --- | --- | --- | --- | --- | --- | --- | --- | --- | --- | --- | --- | --- |
|  | N | Mean  (SD) | Median  IQR | Max | N | Mean  (SD) | Median  IQR | Max | N | Mean  (SD) | Median  IQR | Max |  |
| 5.1 Inpatient | 23 | 27.43  (131.57) | 0  0, 0 | 631.00 | 21 | 20.48  (93.83) | 0  0, 0 | 430.00 | 22 | 19.55  (91.68) | 0  0, 0 | 430.00 | .998 |
| 5.2 Outpatient | 20 | 128.20  (180.17) | 55.50  0, 176.63 | 730.50 | 17 | 100.44  (175.50) | 55.50  0, 123.00 | 691.50 | 18 | 26.00  (51.84) | 0  0, 55.50 | 190.50 | .024 |
| 5.3 Community based day | 23 | 73.39  (233.67) | 0  0, 0 | 952.00 | 21 | 4.57  (20.95) | 0  0, 0 | 96.00 | 21 | 273.98  (698.17) | 0  0,0 | 2,677.50 | .313 |
| 5.4 Other primary and community** | 22 | 342.39  (547.12) | 157.34  72.74, 421.50 | 2,527.50 | 21 | 228.28  (256.81) | 153.00  65.75, 300.00 | 997.50 | 22 | 211.93  (221.05) | 128.12  64.16, 328.22 | 765.00 | .869 |
| 5,4 Home help/ care worker | 23 | 916.33  (4,197.63) | 0  0, 0 | 20,160.00 | 21 | 516.00  (1457.17) | 0  0, 126.00 | 6,300.00 | 22 | 1038.55  (4309.03) | 0  0, 0 | 20,160.00 | .317 |
| Total | 19 | 1611.20  (4,662.95) | 306.00  154.56, 730.50 | 20,600.00 | 17 | 1020.14  (1522.37) | 607.50  228.00, 967.25 | 6,376.00 | 17 | 1828.29  (4949.58) | 276.00  66.00, 504.32 | 20,236.50 | .193 |
| Total plus intervention costs | 19 | 1611.20  (4,662.95) | 306.00  154.56, 730.50 | 20,600.00 | 17 | 1310.14  (1522.37) | 897.50  518.00,1257.25 | 6666.00 | 17 | 2100.29  (4949.58) | 548.00  338.00,776.32 | 20,508.50 | .006 |

** Excludes home help / care worker

| **T2 (16 to 28 weeks)** | **A: TAU** | | | | **B: GiVE** | | | | **C: SC** | | | | **Kruskal Wallis test p** |
| --- | --- | --- | --- | --- | --- | --- | --- | --- | --- | --- | --- | --- | --- |
|  | N | Mean  (SD) | Median  IQR | Max | N | Mean  SD | Median  IQR | Max | N | Mean  SD | Median  IQR | Max |  |
| 5.1 Inpatient | 15 | 42.07  (162.92) | 0  0, 0 | 631.00 | 12 | 107.50  (372.39) | 0  0, 0 | 1290.00 | 15 | 0  (0) | 0  0, 0 | 0 | .550 |
| 5.2 Outpatient | 13 | 98.31  (133.85) | 55.50  0, 190.50 | 405.00 | 9 | 75.83  (82.50) | 55.50  0, 135.00 | 246.00 | 14 | 48.75  (63.12) | 27.75  0, 75.38 | 190.50 | .692 |
| 5.3 Community based day | 15 | 517.80  (1602.91) | 0  0, 135.00 | 6240.00 | 12 | 379.71  (921.46) | 0  0, 7.92 | 2880.00 | 15 | 366.80  (781.54) | 0  0, 288.00 | 2304.00 | .993 |
| 5.4 Other primary and community** | 15 | 233.14  (249.97) | 153.00  50.40, 264.00 | 780.00 | 12 | 182.40  (223.84) | 78.72  23.78, 354.35 | 618.25 | 14 | 238.60  (172.45) | 182.93  101.85, 320.31 | 657.63 | .318 |
| 5,4 Home help/ care worker | 15 | 3.73  (9.85) | 0  0, 0 | 28.00 | 12 | 0  (0) | 0  0, 0 | 0 | 15 | 1344.00  (5205.29) | 0  0, 0 | 20160.00 | .436 |
| Total | 13 | 888.78  (1779.15) | 286.50  121.34, 918.75 | 6681.63 | 9 | 952.09  (1291.78) | 201.75  83.19, 2060.83 | 3509.25 | 13 | 2232.96  (6157.36) | 315.75  190.88, 799.25 | 22620.90 | .776 |
|  |  |  |  |  |  |  |  |  |  |  |  |  |  |
| **Total 0-28 weeks** | 12 | 1418.03  (1938.36) | 839.70  360.47, 1418.17 | 7176.12 | 7 | 1866.90  (1604.85) | 1089.50  590.68, 3757.25 | 4406.75 | 11 | 5581.36  (12677.58) | 852.00  656.90, 3551.00 | 43129.40 | .536 |

** Excludes home help / care worker

***Table 4: Changes in numbers of medications***

| **Period** | **Arms** | **Increase by 2** | **Increase by 1** | **No change** | **Decrease by 1** | **Decrease by 2** | **Decrease by 3** | **Total** |
| --- | --- | --- | --- | --- | --- | --- | --- | --- |
| **T0 - T1** | **A: TAU** | 0 | 2 | 9 | 4 | 2 | 0 | **17** |
|  | **B: GiVE** | 1 | 2 | 8 | 2 | 1 | 1 | **15** |
|  | **C: SC** | 0 | 2 | 13 | 1 | 0 | 1 | **17** |
|  |  | **1** | **6** | **30** | **7** | **3** | **2** | **49** |
| **T1 - T2** | **A: TAU** | 0 | 0 | 9 | 0 | 0 | 1 | **10** |
|  | **B: GiVE** | 0 | 0 | 5 | 0 | 0 | 0 | **5** |
|  | **C: SC** | 0 | 0 | 4 | 2 | 1 | 0 | **7** |
|  |  | **0** | **0** | **18** | **2** | **1** | **1** | **23** |
| **T0 - T2** | **A: TAU** | 0 | 2 | 4 | 4 | 0 | 1 | **11** |
|  | **B: GiVE** | 0 | 0 | 4 | 1 | 0 | 0 | **5** |
|  | **C: SC** | 0 | 1 | 2 | 2 | 1 | 1 | **7** |
|  |  | **0** | **3** | **10** | **7** | **1** | **2** | **23** |

***Table 5: Quality of Life, EQ-5D-5L***

| **EQ-5D-5L**  **Range 0 – 1.00(best)** | **T0 Baseline** | | **T1 Week 16** | | **T2 Week 28** | | **EQ-5D-5L Mean differences** | |
| --- | --- | --- | --- | --- | --- | --- | --- | --- |
|  | **N** | **Mean** | **N** | **Mean** | **N** | **Mean** | **T0 – T1** | **T0 – T2** |
| **A: TAU** | 27 | .419 | 23 | .455 | 16 | .499 | .0417 | .0534 |
| **B: GiVE** | 26 | .581 | 21* | .595 | 11 | .621 | .0029 | .0477 |
| **C: SC** | 26 | .573 | 23 | .586 | 16 | .538 | .0189 | -.0466 |
| **Total** | 73 |  | 67* |  | 45 |  |  |  |
|  |  |  |  |  |  |  |  |  |
| **Mean QALYs gained** | **T0-T1**  **(T0+T1)/2 x 16 weeks** | | **T1-T2**  **(T1+T2)/2x 16 weeks** | | **Regression modelling: the increment in QALYs, compared to TAU, controlling for baseline utility** | | | |
| **A: TAU** | 0.134 | | 0.258 | | **T0 – T1** | **B. GIVE** | .004 | Not significant |
| **B: GiVE** | 0.183 | | 0.350 | |  | **C. SC** | .005 | Not significant |
| **C: SC** | 0.179 | | 0.309 | | **T0 – T2** | **B. GIVE** | .041 | Not significant |
|  |  |  |  |  |  | **C. SC** | .002 | Not significant |

* n=22 participants returned at T1, but one respondent had no EQ-5D-5L

***Appendix: Unit costs used in the health economic analysis***

|  |  | **Source: *Curtis L, Burns A. Unit costs of health and social care 2019. University of Kent.*** [***www.pssru.ac.uk***](http://www.pssru.ac.uk) | |
| --- | --- | --- | --- |
|  |  | **N** | **Unit cost and details** |
| In patient, hospital service | Acute psychiatric inpatient ward: Admissions (Days) | 65 | P36, 2.1. National Reference Costs for Mental Health services NHS Improvement 2017/8 uprated to 2018/9 with NHS cost inflation index: Mental health care cluster £430 per bed day |
|  | Emergency / crisis centre: Admissions (Days) | 65 | As acute psychiatric inpatient above |
|  | General medical ward: Admissions (Days) | 65 | P82, 7.1 National Reference Costs NHS Improvement 2016/17 uprated to 2017/18 with NHS cost inflation index. Non elective inpatient stays (short) £631 – 3 days or less |
| Out patient hospital service | Psychiatric outpatient visit: Appointment | 66 | P150, Section 14. Consultant psychiatrist. Cost per working hour £111. Assume 30 minutes per appointment |
|  | Other hospital outpatient visit (including A&E): Appointments | 55 | P82, 7.1 National Reference Costs NHS Improvement 2016/17 uprated to 2017/18 with NHS cost inflation index. Weighted average of all outpatient appointments £135. Assume zeros for missing |
|  | Day hospital: Day attendance | 66 | As day care below |
| Community based day services | Community mental health centre: Attendances | 66 | As day care below |
|  | Day care centre: Attendances | 66 | P38 (P39) LA (private/voluntary sector) day care for adults requiring mental health support age 18-64: £9.30 (£9) per client hour; £32 per client session lasting 3.5 hours. Assume 1 attendance = 1 session |
|  | Group therapy: Attendances | 66 | P82, 7.1 National Reference Costs for hospital community services NHS Improvement 2016/17 uprated to 2017/18 with NHS cost inflation index. Group sessions with physiotherapist £46; occupational therapist £70; speech and language therapist £119; dietician £86. No data on group psychological therapy so used £119 (top price) as psychologists usually expensive (on professional advice). |
|  | Sheltered workshop: Attendances | 66 | As day care above |
|  | Specialist education: Attendances | 66 | As day care above |
| Other primary and community care,  Contacts | Psychiatrist | 66 | As consultant psychiatrist above |
|  | Psychologist | 66 | P159 – cost per working hour, assume 60 minutes per contact  Band 5 Clinical Psychologist Assistant Practitioner higher, counsellor entry level £34 (GIVE and SC interventionists); Band 8 Clinical Psychologist Manager/Consultant £65 for service use. |
|  | Primary care physician | 66 | P120, 10.3b, GP. £39 per surgery consultation lasting 9.22 minutes |
|  | District nurse | 66 | P117, Cost per hour of patient related work, Band 6 £84, assume 30 minutes per contact |
|  | Community psychiatric nurse/ case manager | 66 | Band 6 nurses, assume 60 minutes per contact |
|  | Social worker | 66 | P130, 11.1. Social worker adult services £51 per hour, assume 60 minutes per contact |
|  | Occupational therapist | 66 | P113 Band 6 OT Specialist cost per working hour £45, assume each contact 1 hour |
|  | Home help/ care worker | 66 | P134, 11.5 Home care worker, social service, face to face weekday day, £28 per hour. Assume 30 minutes per contact |
